# Negative Supercoiling Creates Single-Stranded Patches of DNA That Are Substrates for AID–Mediated Mutagenesis

**DOI:** 10.1371/journal.pgen.1002518

**Published:** 2012-02-09

**Authors:** Jahan-Yar Parsa, Shaliny Ramachandran, Ahmad Zaheen, Rajeev M. Nepal, Anat Kapelnikov, Antoaneta Belcheva, Maribel Berru, Diana Ronai, Alberto Martin

**Affiliations:** 1 Department of Immunology, University of Toronto, Toronto, Canada; 2 Howard Hughes Medical Institute, Department of Molecular, Cellular, and Developmental Biology, University of Colorado at Boulder, Boulder, Colorado, United States of America; University of Washington, United States of America

## Abstract

Antibody diversification necessitates targeted mutation of regions within the immunoglobulin locus by activation-induced cytidine deaminase (AID). While AID is known to act on single-stranded DNA (ssDNA), the source, structure, and distribution of these substrates *in vivo* remain unclear. Using the technique of *in situ* bisulfite treatment, we characterized these substrates—which we found to be unique to actively transcribed genes—as short ssDNA regions, that are equally distributed on both DNA strands. We found that the frequencies of these ssDNA patches act as accurate predictors of AID activity at reporter genes in hypermutating and class switching B cells as well as in *Escherichia coli*. Importantly, these ssDNA patches rely on transcription, and we report that transcription-induced negative supercoiling enhances both ssDNA tract formation and AID mutagenesis. In addition, RNaseH1 expression does not impact the formation of these ssDNA tracts indicating that these structures are distinct from R-loops. These data emphasize the notion that these transcription-generated ssDNA tracts are one of many *in vivo* substrates for AID.

## Author Summary

Creating an effective antibody-mediated immune response relies on processes that create antibodies of high affinity and of different functions in order to clear pathogens. Activation-induced cytidine deaminase (AID) is an essential B cell–specific factor that is known to initiate these processes by deaminating dC on single-stranded DNA of actively transcribed genes. AID has also been implicated in deaminating dC at non-antibody genes, resulting in the disregulation of genes that may lead to B cell–related cancers. Until now, it has remained unknown what the source, structure, and distribution of the single-stranded DNA is that AID acts upon. By using a novel assay that allows direct detection of single-stranded DNA within intact cell nuclei, we observed patches of single-stranded DNA that are strongly correlated to the preferred activity of AID. Furthermore, we find that the activity of AID and single-stranded DNA patch formation can be enhanced by negative supercoiling of the DNA, which is a typical consequence of transcription. These findings allow us to better understand how AID is recruited to and mutates antibody genes as well as other genes implicated in cancers of B cell origin.

## Introduction

The generation of high affinity antibodies is an important feature of the adaptive immune response to pathogens. The iterative process of somatic hypermutation (SHM) introduces mutations into the DNA encoding the variable (V) region of the antibody molecule that ultimately confer higher affinity for pathogen-derived antigen. Immunoglobulin (Ig) genes also undergo class switch recombination (CSR), which creates antibodies of different isotypes with distinct effector functions. The importance of these secondary diversification processes is demonstrated in hyper-IgM type II patients that lack these diversification processes and are thus immunocompromised [1].

Activation-induced cytidine deaminase (AID) initiates secondary diversification of Ig genes by deaminating dC within the V-region to initiate SHM and gene conversion, or within switch regions to initiate CSR [2]. The deamination event is then processed by downstream repair pathways that result in the mutagenic processes during SHM and in the production of double-stranded DNA (dsDNA) breaks during CSR. The mechanism by which AID preferentially deaminates dC in Ig genes rather than other loci has still not been fully elucidated, although recent reports have revealed that proteins involved in transcription play key roles in AID-targeting. Spt5, an elongation factor that directly interacts with the RNA polymerase, has been found to bind and target AID to mutating genes [3]. Furthermore, through *in vitro* SHM studies, the RNA exosome, a complex of factors that specialize in an array of diverse RNA processing events, has been shown to direct AID to both template and non-template strands of DNA [4]. Indeed, the role of transcription in SHM has long been suspected [5]–[9].

In the case of SHM of Ig genes, AID deaminates dC in both DNA strands, as evidenced by sequencing of V-region DNA [10], [11] and the finding that AID induces dUs equally on both strands of the DNA [12]. In the case of CSR, which requires a double strand break [13], AID is also thought to act on both strands of the Ig switch (S) region. That is, a plausible model for generating the double strand break envisages that AID will sometimes deaminate dCs that are close to each other but in opposite strands. These deaminations create G:U mismatches that are substrates for mismatch repair and UNG, which introduce single strand breaks close by in each strand, thus effectively making a double-strand break.

It is well established that AID directly deaminates dC *only* within single-stranded regions [14]–[17]. Our previous work showed that both strands of the Ig heavy chain V-region DNA of B cell lines and *ex vivo* mouse B cells contains single stranded patches and that these patches are more frequent in the V-region than in other genes [18]. Nonetheless, much work has been undertaken to unveil how both strands of the V and switch regions would be rendered single-stranded. Crystallographic studies have revealed that there is little exposed single-stranded DNA (ssDNA) in RNA polymerase II transcription bubbles [19], [20]. Likewise, the structure of RNA polymerase II complexed with Spt5 was recently solved, and again, ssDNA was shown to exist solely within the RNA polymerase II complex [21], [22]. In addition, because RNA polymerase II has been found to transcribe the Igμ gene in only the sense direction [23], the bottom (or template) DNA strand should be protected from deamination by nascent RNA produced by the elongating complex. A similar conundrum exists for CSR. That is, although some studies suggest that AID mediates CSR by mutating ssDNA exposed by RNA-DNA hybrids (i.e. R-loops; [24]) on the top strand DNA of the switch region, this model does not explain how AID would mutate the bottom DNA strand within R-loops to produce a dsDNA break. AID may access the bottom strand of the switch region through several mechanisms. One mechanism involves exposure of the bottom strand of a transcribed switch region by AID-directed ExoI-induced excision of the top DNA strand which would then lead to a second round of AID attack on the bottom strand [25]. A different explanation for how AID might act on both DNA strands is suggested by the observation that purified AID mutates supercoiled plasmid DNA on both strands *in vitro* [26]. That is, it is possible that substrate accessibility to AID is mediated by the supercoiling of DNA that is caused by transcription, which may be enhanced by a stalled transcription complex by Spt5 [3], and/or the RNA exosome [4].

As described in the present report, we have sought to test the role of supercoiling in rendering both strands of DNA accessible to AID. In previous work, we used sensitivity to bisulfite-mediated deamination to detect regions of ssDNA. We showed that in hypermutating B cells, the Ig V-region is enriched for patches of ssDNA compared to genes that are not mutated by AID [18]. In the present study, we have used a similar assay and found that actively transcribed genes are enriched for ∼7-nucleotide ssDNA patches, both in mammalian cells and in *E.coli*. These patches are present on both DNA strands. Furthermore, the frequency of these patches is an accurate predictor of AID activity at reporter substrates in mammalian cells and in *E.coli*. These ssDNA patches are not caused by RNA-DNA hybrids (i.e. R-loops), but instead, are produced by DNA supercoiling caused by transcription elongation. As described here, our results suggest one simple and specific method for how transcription renders DNA accessible to AID.

## Results

### ssDNA Frequency Correlates with Somatic Hypermutation Rates at a Transgene

We previously described an assay for detecting ssDNA in cells, based on the capacity of sodium bisulfite to convert dC to dU in single stranded but not double stranded DNA [18]. In this assay, fixed nuclei are exposed to sodium bisulfite, after which DNA is isolated and sequenced to identify regions in which dC was converted to dU. By carrying out the sodium bisulfite reaction in nuclei, we aimed to preserve protein-DNA interactions as they exist *in vivo*, as well as regions of ssDNA that would otherwise be disrupted during nucleic acid isolation. We have used this assay on both *ex vivo* mouse B cells and on Ramos cells - an immortalized centroblast-like cell line which undergoes constitutive SHM in culture.

Continuing this study, we now show that the V-region has a higher ssDNA frequency over other regions within the Ig locus (Figure S1A). Although sodium bisulfite and AID produce the same biochemical transaction (i.e. deamination of dC), ssDNA frequency within the V-region is not reduced in AID-deficient Ramos cells (Figure S1B) or in AID−/− mice [18] indicating that the tracks of deaminated cytidines (i.e. dU) that we observed in B cells are caused by sodium bisulfite. Inasmuch as AID acts on ssDNA, the correlation between the frequencies of SHM and ssDNA patches at different genomic regions suggested that by identifying the molecular basis of the ssDNA patches we might elucidate how AID is recruited in SHM in this context.

**Figure 1:**
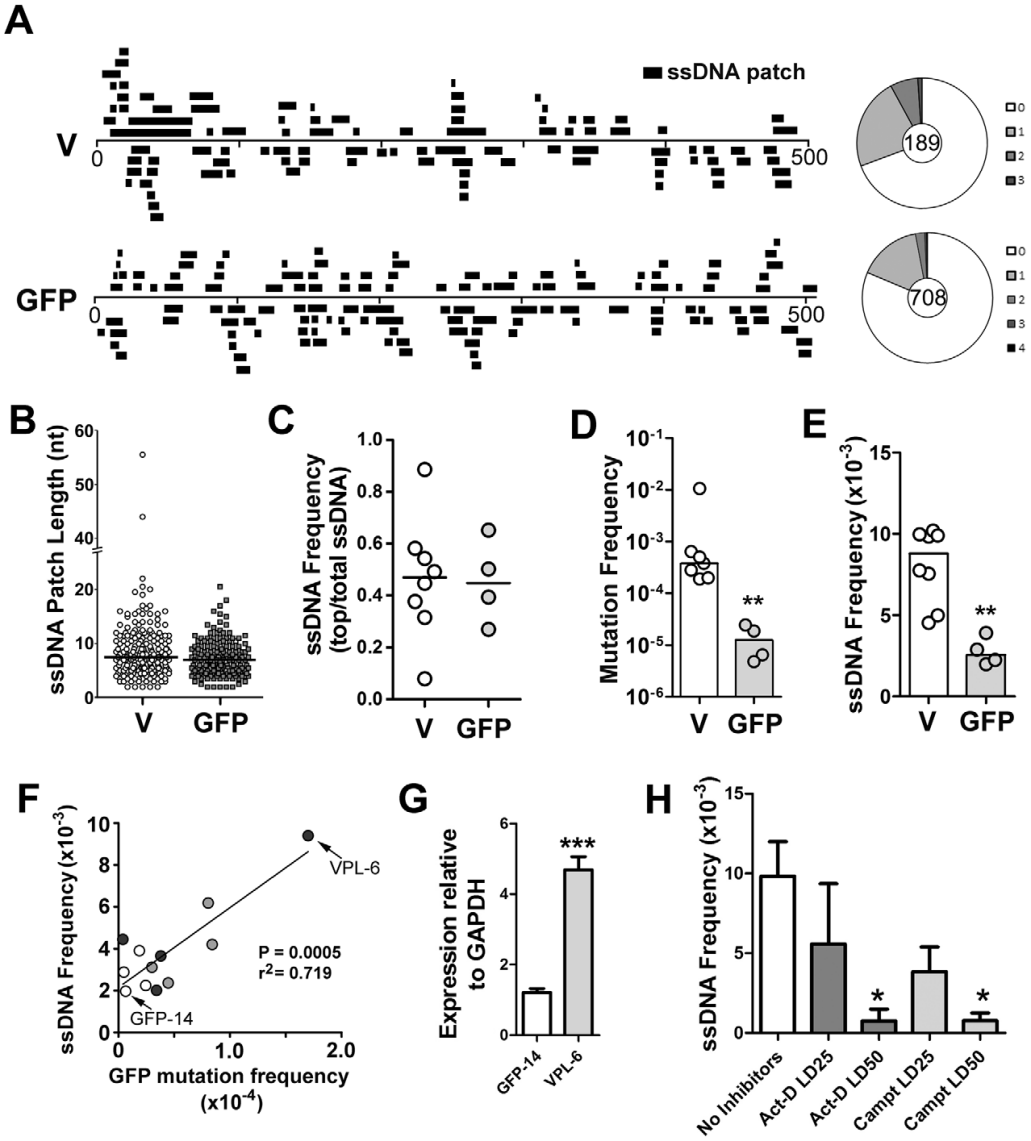
ssDNA Patches in the V-Region and GFP Transgene in Ramos Cells. A) Location and lengths of patches identified in the V-region and GFP transgene. Patches are depicted as black bars and distributed to depict top- or bottom-strandedness. Wagon-wheels depict the number of ssDNA patches per sequence for each gene. B) Cluster plot of patch lengths of ssDNA identified at the V region and GFP genes. Black line depicts the median patch size. C) Strand bias as an expression of ssDNA frequencies on the top strand divided by total ssDNA frequencies for each Ramos clone at the V-region and GFP transgene. A value of 0.5 suggests no strand bias. D) Mutation frequencies at the V-region and GFP genes for individual clones are reported. Statistical analysis was performed using the Mann-Whitney test (** = P = 0.0061). Values for V-region mutation frequencies were obtained from 2 Ramos clones for this study, as well as 5 Ramos clones from previous analyses [28], [65]. E) ssDNA frequencies at the V-region and GFP genes for individual clones. Statistical analyses were performed using the Mann-Whitney test (** = P = 0.0013). F) Linear regression analysis depicting ssDNA frequencies plotted against mutation frequencies for the GFP gene for 12 individual clones (GFP = open circles; VPS GFP = light grey circles; VPL GFP = dark grey circles; see Figure S2C). X and Y axes are plotted as linear values. G) Quantitative RT-PCR analyses for GFP expression was performed on the clones in which the GFP gene mutated at high rates (Figure S2C; VPL-6) or low rates (Figure S2C; GFP-14). See also Figure 1F. The expression of GFP was plotted relative to GAPDH expression. The relative GFP expression levels of one replicate of clone GFP-14 was set to 1. Statistical analysis was performed using the Student's t-test (*** = P = 0.0009). H) ssDNA frequencies at the V-region in Ramos cells treated with the transcription elongation inhibitor, actinomycin D, or the topoisomerase I inhibitor, camptothecin, for 24 hrs with varying concentrations of inhibitors (LD25 and LD50). Act-D = Actinomycin D; Campt = Camptothecin. Statistical analysis was performed using the Student's t-test (* = P = 0.0115 comparing ssDNA frequencies in Act-D and untreated cells; * = P = 0.0108 comparing ssDNA frequencies in Campt and untreated cells).

As a first step in this analysis, we sought to measure whether the frequency of ssDNA patches correlates with mutation rates of the same genetic sequence. We measured these features in two DNA regions of our Ramos cell lines: the heavy chain V region and a GFP reporter gene (Figure 1A). For this analysis, we utilized a provirus that harbours a GFP gene containing a nonsense codon (TAG) located within a preferred target motif for AID (i.e. WRC), such that mutations (revertants) are detected as GFP-positive cells [27] (Figure S2A). This retrovirus does not contain any obvious Ig sequence. The retrovirus was stably integrated into the Ramos genome at a single copy (data not shown), and mutation frequencies at the GFP gene were measured by fluctuation analysis [28]. Using both AID-proficient and AID-deficient Ramos cells we prepared multiple cell lines bearing independent GFP proviruses, and as expected, mutation was greatly decreased in AID-deficient Ramos cells (Figure S2B). Figure 1A shows the representation of all the accumulated data on the actual location and length of each patch of ssDNA in the V-region and GFP genes. Wagon-wheels are depicted for each gene showing the number of ssDNA patches present per sequence analyzed. The majority of sequences which harbour ssDNA contain only one patch per sequence (Figure 1A). Our analysis thus showed that both strands of both V-region and GFP genes included patches of ssDNA. As shown previously [11], this finding correlates relatively well with the fact that AID mutates both strands at approximately equal frequencies (Figure S2D; see below for further discussion).

In order to compare results for different cell lines, different genes and different conditions, we have defined several summary measurements. Median ssDNA patch length is deduced from a cluster plot of the observed patch lengths (Figure 1B); ssDNA strand bias (Figure 1C) is defined as the fraction of the total number of nucleotides in ssDNA patches present on the top strand; ssDNA density (Figure 1E) is defined as the total number of nucleotides in single stranded patches divided by the total number of nucleotides examined for each gene in each cell line. As shown in Figure 1, the median length of these patches was similar in the V-region and GFP DNA (i.e. 7 nucleotides; Figure 1B), and, like the V-region, the patches within the GFP gene were equally distributed on both top and bottom strands (Figure 1C). Mutation rates at the GFP gene were ∼30-fold lower than at the V-region (Figure 1D), while the frequency of ssDNA at the GFP gene was ∼3.5 fold lower than at the V-region (Figure 1E), providing initial evidence of a link between SHM and ssDNA patch frequency.

To test whether the ssDNA frequency at the GFP gene correlates with SHM rates we required Ramos cell lines that mutate the GFP gene at differing rates. One source of such material was to use independently generated proviruses, which, as found in similar systems, had different mutation rates (Figure S2C). We also sought to use cell lines in which the difference in mutation frequency was genetically determined. Based on a previous report that the V-region promoter has an element that can enhance SHM [29], we tested whether incorporation of the Ramos V-region promoter enhances SHM of the GFP transgene. Indeed, we found that the GFP reversion frequency can be enhanced ∼5-fold by placing a 245 bp or a 1.1 kb segment of the Ramos V-region promoter upstream of the GFP gene (Figure S2A and S2B; VPS and VPL, respectively).

We then measured the occurrence of ssDNA patches in selected cell lines and defined a simple parameter, frequency of ssDNA. Using 12 independent transfectants in which the GFP gene is mutating at rates ranging from 0.4 to 17×10^−5^ mutations/base pair/generation (Figure S2C), we found that there was a strong correlation (r^2^ = 0.719, p = 0.0005) between ssDNA frequency and mutation frequency at the GFP gene (Figure 1F). Furthermore, as transcription rates correlate with AID activity, we compared transcript levels of the GFP transgene in the Ramos transfectants that produced both the highest and lowest frequencies of ssDNA/mutations at this site (VPL-6 and GFP-14, respectively: Figure 1F). As seen in Figure 1G, transcript levels correlate with both ssDNA patch formation and SHM frequency, supporting a role for transcription in this process. Moreover, ssDNA patch density is reduced in a dose dependent manner by inhibiting transcription elongation with actinomycin D (Figure 1H), as expected from our previous observation that α-amanitin, which inhibits transcription initiation, ablates the presence of ssDNA [18]. Interestingly, we also observed a dose dependent reduction is ssDNA with the use of the topoisomerase I inhibitor, camptothecin (Figure 1H). While this effect of camptothecin has an indirect inhibitory effect on transcription elongation, camptothecin inhibits topoisomerase I and alters the local superhelicity of the DNA. Thus, the reduction in ssDNA frequency observed using camptothecin may be independent of its effects on transcription elongation. Although these data suggest that transcription elongation is responsible for the generation of ssDNA patches, the effects of these inhibitors on ssDNA formation may be due to secondary or indirect effects, and thus caution must be exercised when interpreting this data. Collectively, these data show that the frequency of ssDNA correlates both with transcription and with AID mutation rates within the same genetic sequence, thus providing circumstantial evidence that these bisulfite-accessible ssDNA patches are substrates for AID.

### ssDNA Patches within the Murine 5′μ Switch Region Correlate with the Requirements of Class Switch Recombination

Previous work has suggested that CSR is mediated by AID-deamination of R-loops within switch regions [24], [30]. R-loops are RNA:DNA hybrids generated by the RNA polymerase II complex and expose the top strand for AID-mediated attack [24], [30]. However, the current model for AID-induced CSR requires the deamination of both DNA strands within switch regions leading to the generation of a staggered dsDNA break [31]–[33]. Because R-loops were previously identified using purified nucleic acids treated with bisulfite [24], [30], we reasoned that ssDNA patches may have been disrupted during this purification process, as we previously found for the V-region [18].

**Figure 2:**
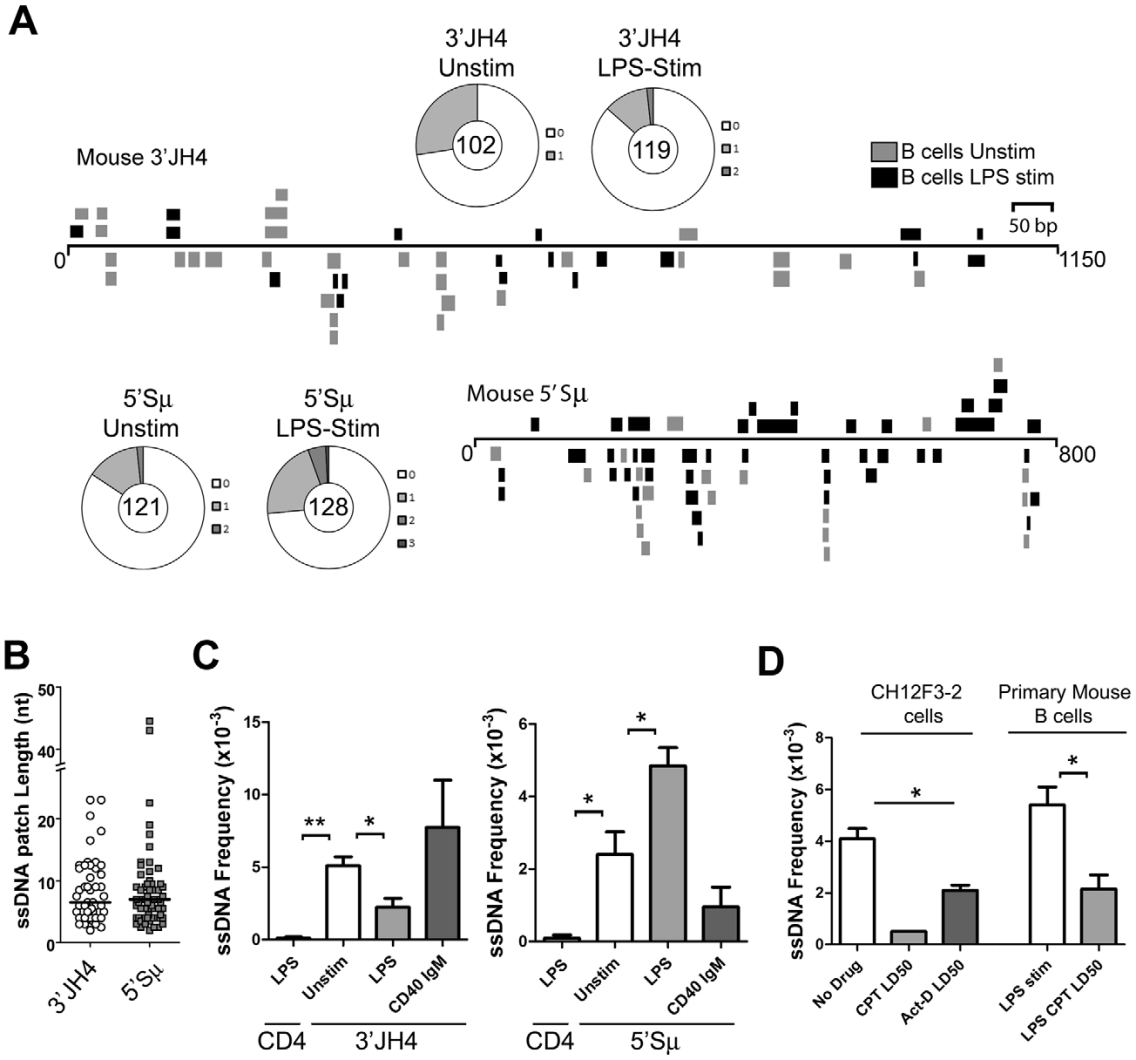
ssDNA Frequencies during Induction of CSR in *Ex Vivo* Murine B Cells. A) ssDNA patches depicted 3′ of the JH4-region and 5′ of the μ switch region in *ex vivo* mouse B cells. ssDNA patches observed in unstimulated B cells (grey box), and LPS-stimulated B cells for 48 hours (black box) are shown. Patches are depicted as bars and are distributed to depict top- or bottom-strandedness. Wagon-wheels depict the number of ssDNA patches per sequence for both regions examined under unstimulated or LPS stimulated conditions. B) ssDNA patch lengths of the mouse V-region and 5′μ switch region. Median patch lengths depicted by a black line. C) ssDNA frequencies observed within murine *CD4* and the 3′JH4 region (left panel) and 5′Sμ (right panel) in unstimulated (open bar), LPS-stimulated (grey bar), and IgM and α-CD40 stimulated (dark grey bars) *ex vivo* mouse B cells. Statistical analyses were performed using the Student's t-test (Left Panel: ** = P = 0.002 comparing ssDNA frequencies at the CD4 gene and unstimulated 3′JH4 region; * = P = 0.021 comparing ssDNA frequencies at the V-region between unstimulated and LPS-stimulated B cells; Right Panel: * = P = 0.039 comparing ssDNA frequencies at the CD4 gene and the unstimulated 5′Sμ * = P = 0.036 comparing ssDNA frequencies at the 5′Sμ region between unstimulated and LPS-stimulated B cells). D) ssDNA frequencies at the 5′μ switch in CH12F3-2 cells and primary mouse B cells that were untreated or treated with Camptothecin (Campt) or Actinomycin D (Act-D) for 24 hrs. Since no ssDNA patches were observed in the Campt-treated CH12F3-2 cells, the data is presented as if one dC were converted to dT Statistical analysis were performed using the Student's t-test (* = P = 0.023 comparing CH12F3-2 no drug and Act-D LD50; * = P = 0.026 comparing Primary Mouse B cells LPS stim and LPS CPT LD50).

To test whether ssDNA patches exist on both strands in switch regions, we carried out the bisulfite reaction on purified nuclei from primary murine B cells that had been stimulated to undergo CSR by lipopolysaccharide (LPS) treatment. As shown in Figure 2A, ssDNA patches were identified downstream of the JH4 region as well as immediately upstream of the μ-switch region in mature unstimulated and stimulated B cells. Wagon-wheels depicting the number of ssDNA patches per sequence show that most sequences that harbour ssDNA only contain one ssDNA patch (Figure 2A). Similar to the data obtained in Ramos cells, the patches had a median length of ∼7 nucleotides at both locations (Figure 2B), and were present on both the top and bottom strands with approximately equal frequencies upon LPS-stimulation (Figure S3A). Importantly, we observed no ssDNA patches in a non-transcribed gene, *CD4*, while unstimulated or LPS-stimulated B cells were enriched for ssDNA at the 3′JH4 region, the 5′μ-switch region (Figure 2C). These findings further support the role of transcription in producing ssDNA patches.

Interestingly, LPS-stimulation led to a statistically significant drop in the ssDNA frequency at the 3′JH4 region and a comparable increase in ssDNA frequency at the 5′μ-switch region (Figure 2C). This result was due to a decrease in the percent of sequences harbouring ssDNA patches at the 3′JH4 region and an increase in the percent of sequences harbouring ssDNA patches in the 5′Sμ region (Figure 2A). These findings are consistent with previous results that LPS-stimulation induces CSR, but not SHM, in primary murine B cells [34]. On the other hand, stimulation of *ex vivo* B cells with IgM and α-CD40, which activates B cells but does not induce CSR (data not shown), does not lead to an increase in ssDNA patch formation at the Sμ region (Figure 2C). However, we found that various treatments that stimulate CSR to IgG1, IgG3 and IgA did not lead to an increase in the ssDNA frequency in the 5′ regions of the Sγ1, Sγ3, and Sα regions, respectively (Figure S3B). While we don't know the reason for this result, it is consistent with the finding that conditions that stimulate CSR to IgG3 and IgG1 do not lead to AID-induced mutations [35] or to AID-induced dsDNA breaks [36] in the Sγ3 region, as opposed to the Sμ region [35], [36].

We next tested whether the ssDNA patches at the Sμ region could be reduced using the topoisomerase I inhibitor, camptothecin, as well as a transcription elongation inhibitor, actinomycin D, as was observed for SHM. In fact, camptothecin, which reduces switch region breaks and CSR in the CH12F3-2 murine cell line [37], also disrupts ssDNA patch formation in CH12F3-2 cells as well as in primary mouse B cells stimulated with LPS (Figure 2D) suggesting that the reduction in AID-induced switch region breaks might be due to a reduction in ssDNA patches. Treating CH12F3-2 cells with actinomycin D also led to a decrease in the ssDNA frequency at the 5′Sμ region (Figure 2D). Once again, the effects of these inhibitors on ssDNA formation may be due to secondary or indirect effects, and thus caution must be exercised when interpreting this data. Nevertheless, these data suggest that the bisulfite-accessible patches within the Ig locus in primary murine B cells are 1) caused by transcription elongation and 2) might allow access to AID on the bottom DNA strand within the switch region.

### Mutation Frequency Somewhat Correlates with ssDNA Frequency at Non-Ig Genes

**Figure 3:**
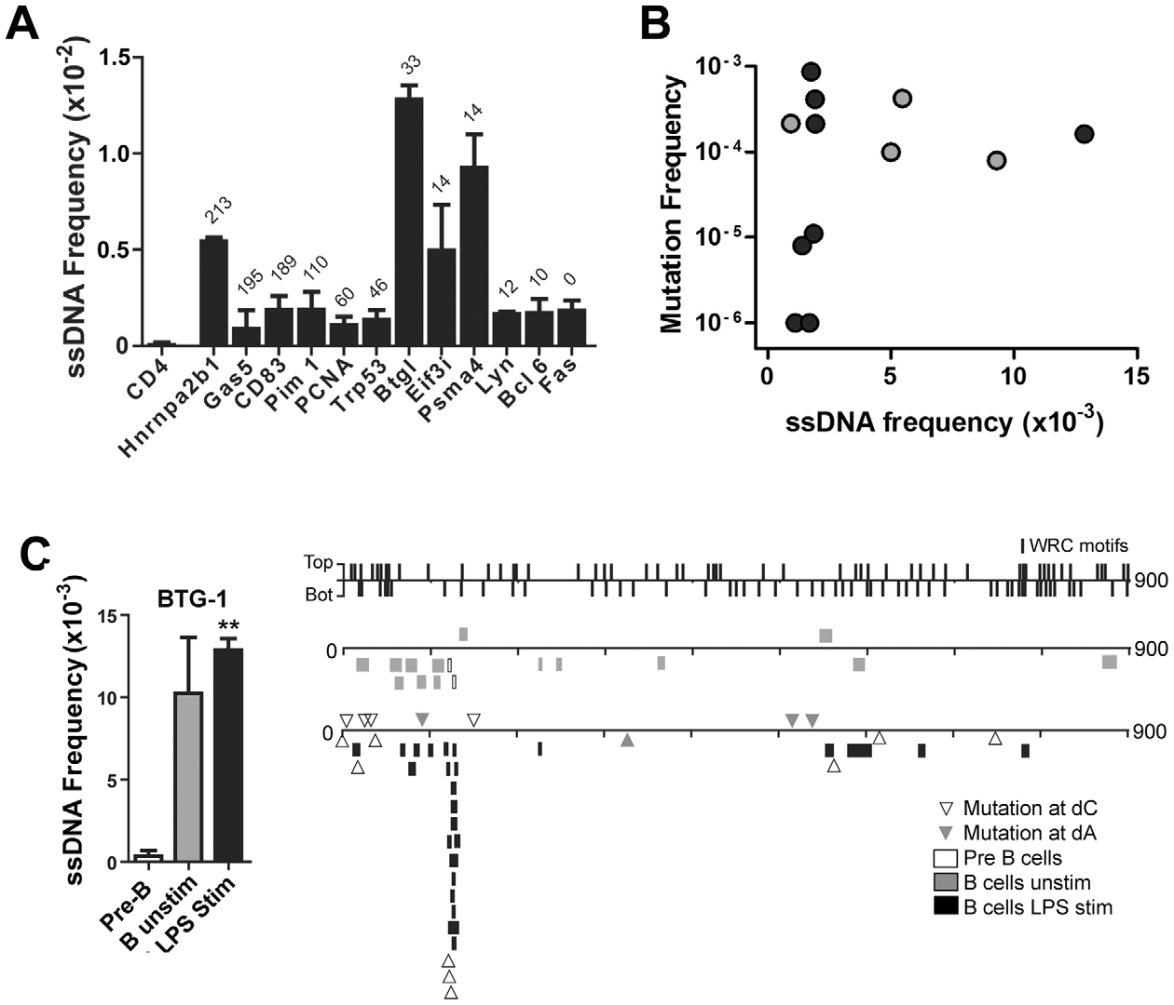
ssDNA Frequencies at Non-Ig Sequences in LPS-Stimulated *Ex Vivo* Murine B Cells. A) ssDNA frequencies at the non-transcribed *CD4* gene, and other indicated genes that are transcribed in B cells. The genes are ordered from Spt5^hi^ (left) to Spt5^lo^ (right) and the number of αSpt5 TPM (tags per million sequences) obtained from Pavri *et al.* (see Table S3 in [3]) is indicated above each gene, which correlates with Spt5 occupancy. B) ssDNA frequency plotted against the mutation frequency of each gene examined in (A). Mutation frequencies for each gene were obtained from Liu *et al.* [38] (black symbols) and Pavri *et al.* [3] (grey symbols). C) Left Panel: ssDNA frequencies at the *Btg1* gene in pre-B cells, unstimulated B cell and LPS-stimulated B cells. Statistical analysis were performed using the Student's t-test (** = P = 0.0039 comparing ssDNA frequencies at the *Btg1* gene in pre-B cells and LPS-stimulated B cells). Right Panel: ssDNA patches depicted along the *Btg1* gene with unique AID-induced mutations. ssDNA patches observed in pre-B cells (open box), unstimulated mature B cells (grey box), and LPS-stimulated B cells (black box) are shown. Unique point mutations depicted at dC (open triangles) or dA (closed triangles) are shown and were obtained from Liu *et al.* [38]. The location and strand distribution of WRC motifs are depicted as lines along the *Btg1* gene at the top of this panel.

A recent report shows that Spt5 density at a gene predicts AID mutability [3]. Although ssDNA does not occur within the Spt5-RNA polymerase II complex [21], [22], it may be enriched near this complex. Hence, we examined whether the frequency of ssDNA patches correlates with Spt5 density in LPS-stimulated primary mouse B cells. As mentioned above, we found that the *CD4* gene does not have any detectable ssDNA patches (Figure 3A). By contrast, ssDNA patch formation was detectable in all transcribed genes that we examined, and was higher in some genes (i.e. *Btg1* and *Psma4*) than what we found within Ig genes in primary murine B cells (Figure 2C). Although we did not observe a correlation between Spt5 density and ssDNA patch frequency (Figure 3A), we found that the presence of ssDNA patches is a common feature of transcribed genes. However, we did observe a correlation between ssDNA frequency and mutation frequency in some of these genes (Figure 3B). That is, 4 out of 4 genes that have a ssDNA frequency above 0.005 have mutation frequencies above 7.5×10^−5^, while only 4 out of 8 genes that have a ssDNA frequency below 0.005 have mutation frequencies above 7.5×10^−5^ (Student's t-test, P<0.05). In fact, closer examination of the *Btg-1* gene, which had the highest frequency of ssDNA of all genes, revealed that mutations at dC reported by Liu *et al.* [38] clustered around a region in the *Btg-1* gene that was enriched in ssDNA patches (Figure 3C). These data indicate that ssDNA patch frequency is a predictor of AID mutability, but also reveals that other factors or properties of a gene are important for AID-induced mutations.

### ssDNA Patches and AID Mutagenesis Are Enhanced by Negative Supercoiling

Transcription has been shown to produce local supercoiling of transcribed DNA [39]; positive supercoiling (increased winding of the DNA) occurs downstream of the transcription complex while negative supercoiling (increased unwinding of the DNA) occurs upstream [40], [41]. Negative supercoiling produces melted DNA [42], [43], and so we hypothesized that negatively supercoiled DNA may be responsible for the ssDNA patches characterized in this report. Interestingly, Storb and colleagues previously showed that AID has increased mutagenic activity on (negatively) supercoiled plasmid DNA *in vitro* [26].

In bacteria, negative supercoiling is relieved by topoisomerase 1 (TopA), and as a result, the DNA of TopA mutant bacteria is more highly supercoiled than in wildtype bacteria [44]. In principle, a comparison of ssDNA and AID-mediated mutation rates in wildtype and TopA mutants might indicate whether negative supercoiling is important for AID activity. Previous work has shown that AID is mutagenic in *E. coli* [7], [14]. We therefore tested whether DNA in *E.coli* has ssDNA patches similar to those seen in mammalian DNA, and if so, whether the density of the patches is higher in the absence of topoisomerase.

**Figure 4:**
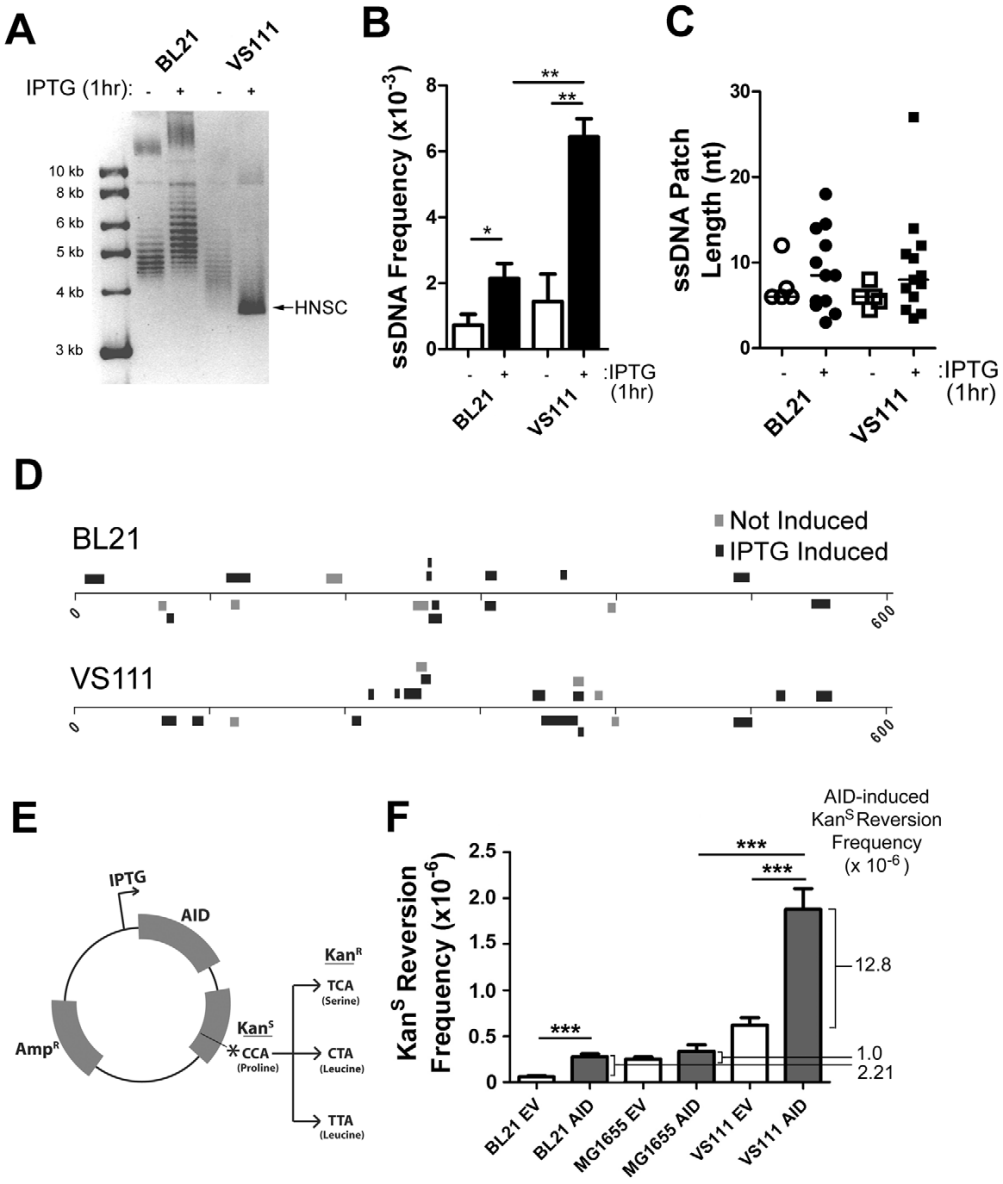
ssDNA Patch Formation and Negative Supercoiling in *E. coli*: Wild-Type BL21(DE3) versus TopA Deficient VS111(DE3). A) p-GEX5.3-hAID(T27N) plasmid topology was determined for uninduced and 1 hr IPTG-induced bacterial strains by chloroquine agarose gel analysis for plasmids derived from wildtype BL21(DE3) and TopA-deficient VS111(DE3) bacterial strains. Highly-negative supercoiled DNA (HNSC) runs faster than its less supercoiled counterpart. B) ssDNA frequencies in uninduced and IPTG-induced bacterial strains. White bars indicate no IPTG treatment, black bars indicated IPTG treatment. Statistical analysis was performed using the Student's t-test (* = P = 0.0446 comparing ssDNA frequencies in uninduced and IPTG-induced BL21 cells; ** = P = 0.0059 comparing ssDNA frequencies in uninduced and IPTG-induced VS111 cells; ** = P = 0.0018 comparing ssDNA frequencies in IPTG-induced BL21 and VS111). C) ssDNA patch lengths in non-IPTG induced (open symbols) and IPTG induced (closed symbols) BL21 and VS111 bacterial strains. D) The location, lengths and strand distribution of ssDNA patches revealed in the p-GEX5.3-hAID(T27N) plasmid by *in situ* bisulfite treatment in uninduced (grey) and IPTG-induced (black) BL21(DE3) and VS111(DE3)[TopA mutant]. E) Schematic of the p-GEX5.3-hAID(WT)KanS vector used for the kanamycin reversion analysis. Kanamycin sensitivity is rendered by a proline (CCA) at position 94 of the kanamycin protein. C to T mutation of either the first or second C of the codon results in a serine or a leucine amino acid, respectively, which confers kanamycin resistance. F) AID-induced mutation frequencies of the kanamycin gene in BL21, MG1655 and TopA-deficient VS111 bacterial strains. Values were calculated after subtracting background mutation frequencies obtained from the mutation analysis of the p-GEX5.3KanS vector controls. EV = empty vector control; AID = AID expression vector (E). Statistical analysis were conducted using the student's two tailed t-test (*** = P = 0.0001 comparing BL21 EV and BL21 AID; *** = P = 0.0006 comparing MG1655 AID and VS111 AID; *** = P = 0.0008 comparing VS111 EV and VS111 AID).

For this purpose we examined the structure of an inducible gene in *E.coli*. Wildtype [BL21(DE3)] and TopA mutant [VS111(DE3)] bacteria were transformed with an IPTG-inducible expression vector, treated with IPTG for 1 hour, and then subjected to a modified version of the *in situ* bisulfite protocol (see Materials and Methods). In this assay, we used an IPTG-inducible AID expression vector that expresses a catalytically dead AID(T27N), although any primary sequence would suffice for this analysis. Supercoiling can be assayed on bacterial plasmids by its mobility in chloroquine gel electrophoresis (see Materials and Methods). As expected, IPTG induction resulted in hyper-negative supercoiling (HNSC) in VS111(DE3) but not in BL21(DE3) controls (Figure 4A) as observed by the strong single banding pattern observed in the IPTG induced VS111(DE3) lane (Figure 4A, lane 4 and Figure S4A). IPTG induction also increased the ssDNA patch density in both strains of *E. coli* (Figure 4B), but the increase was ∼3 fold greater in the TopA mutant. Patch length was not significantly affected by IPTG induction and was similar in both strains to the length that we observed in mammalian cells (Figure 4C) and patches were present on both strands of the DNA (Figure 4D and Figure S4B).

To determine whether increased negative supercoiling also results in increased AID mutagenic activity, we generated an IPTG-inducible AID expression vector that also contains a Kanamycin resistance (Kan^R^) gene with a L94P mutation which results in Kanamycin sensitivity (Kan^S^) (Figure 4E). This mutation exists in a preferential target for AID (TACC is a WRCY motif). Mutation of either of the two cytidines at proline 94, resulting in either a serine or leucine at residue 94, restores Kan^R^ (Figure 4E).

Using the empty vector control, p-GEX5.3KanS, that does not code for AID, we found that the background mutation frequency of the TopA mutant strain VS111 was ∼10-fold higher than the BL21 strain, and ∼2-fold higher than the MG1655(DE3) strain, which is the parental wildtype strain to VS111 (Figure 4F). MG1655(DE3), like the BL21 strain, is not deficient in TopA and thus does not undergo hypernegative supercoiling as observed on a chloroquine gel electrophoresis (Figure S4A). Importantly, we observed a 5.8 to 12.8-fold increase in AID-induced mutation frequency in the VS111 strain (TopA deficient) relative to the BL21 strain and MG1655 strain, respectively (Figure 4F), even though IPTG induction led to similar AID protein levels in each strain (Figure S4C). These results indicate that negative supercoiling induced by transcription creates ssDNA patches and increases AID-mediated mutagenesis. Together with other work showing that AID deaminates dC only in ssDNA, our results argue that one role of transcription in SHM is to create supercoiling, which then generates single-stranded DNA patches that are substrates for AID-mediated deamination.

### RNaseH1 Does Not Impact Bisulfite-Accessible ssDNA Patches

**Figure 5:**
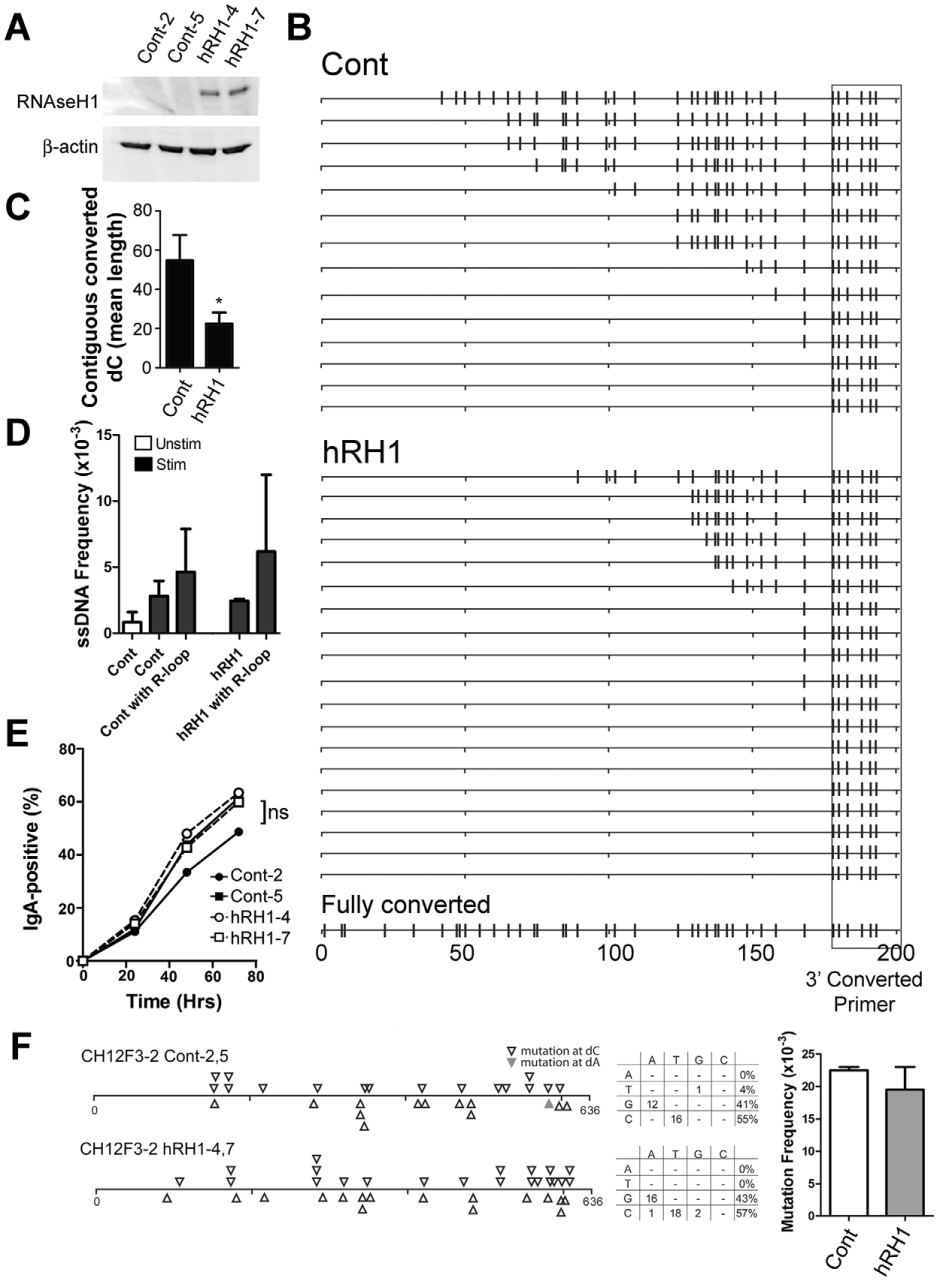
R-Loop Formation, ssDNA Patches and Switching to IgA in WT and RNaseH1-Expressing CH12F3-2 Cells. A) Two CH12F3-2 control clones and two CH12F3-2 clones expressing RNaseH1 (hRH1) transfected with a hRH1 expression vector. Western blots were performed for RNaseH1 and β-actin. B) Switch region R-loop formation in control and RNaseH1-expressing CH12F3-2 cells. Extracted genomic DNA from *in situ* bisulfite treated-CH12F3-2 clones were subjected to PCR amplification using a standard forward primer and a reverse primer that preferentially binds to bisulfite-converted dCs on the top strand 5′ of the μ switch region to specifically amplify bisulfite converted products/R-loops (see Materials and Methods). A hypothetical sequence in which all the dCs are converted to dUs is shown on the bottom for reference. The location of the reverse converted primer is depicted as a box. C) R-loop length as expressed as the mean length of contiguous converted dCs in control and RNaseH1-expressing CH12F3-2 cells. Data derived from B. Statistical analysis were conducted using the Student's two tailed t-test * = P = 0.016. D) ssDNA patch frequency, obtained from amplifying the switch region with unconverted primers, shown as ssDNA frequencies in control and RNaseH1-expressing CH12F3-2 cells before (Unstim) and after CSR-stimulation (Stim and hRH1) [46]. ssDNA frequency also analyzed in association with sequences that harbour R-loops (Cont with R-loop and hRH1 with R-loop). E) CSR to IgA in stimulated control and RNaseH1-expressing CH12F3-2 cells. ns = not significant. F) Mutation analysis in WT and hRH1 CH12F3-2 clones of the 5′Sμ region. Left Panel: Mutations are depicted at dC (opened triangle) or dA (closed triangle) along the 5′Sμ region. Middle Panel: mutation spectrum represented for WT and hRH1 samples. Right Panel: Mutation frequencies for WT and hRH1 samples (no significant difference observed).

Another model for generating ssDNA focuses on the role of R-loops, in which the RNA-DNA hybrid formed in transcription renders the coding strand single-stranded. We tested whether the bisulfite-accessible patches found on both strands in the 5′μ switch region are caused by R-loop formation. We used the CH12F3-2 murine B cell line for this analysis, which is a cell line that can be induced to switch to IgA at high levels [45] and switching is exquisitely sensitive to the protein levels of factors required for CSR such as AID (Figure S5A and S5B: knockdown of AID by 2.4 and 5.8-fold leads to a 2.5 and 5.0-decrease in CSR, respectively) [3], RNF8/RNF168 [46], and DNA Ligase IV [47]. We then tested whether a human RNaseH1 (hRH1) expression vector reduces ssDNA patches in CH12F3-2 cells. hRH1 degrades RNA-DNA hybrids and has been shown to reduce R-loops when expressed in cells [48]. Two stable clones were obtained that overexpressed hRH1 (Figure 5A; clones hRH1-4 and -7). To test whether hRH1 was functional in these cells, we treated nuclei from the control and hRH1-expressing CH12F3-2 clones with the *in situ* bisulfite assay, and amplified R-loops using a 3′primer that binds to C to U converted DNA near the switch region and the standard 5′primer upstream of the 5′μ switch region that was used in Figure 2A. We observed a decrease, but not an ablation, in contiguously converted dC lengths (which likely represent R-loops) in clones expressing hRH1 compared to controls (Figure 5B and 5C) suggesting a decrease in R-loop length and frequency and offering a correlative readout that the hRH1 is active in the transfected cells. In addition, 8/19 (42%) sequences from the hRH1 clones did not have converted sequences 5′ of the primer compared to 3/14 (21%) for controls (Figure 5B) further suggesting that the hRH1 is indeed functional in this system. On the other hand, ssDNA patch frequency was unaffected in hRH1-expressing clones (Figure 5D and Figure S5C–S5E), and RNaseH1 did not affect the proliferative properties of CH12F3-2 (Figure S5F), suggesting that unlike R-loops, the ssDNA patches are not the product of RNA-DNA hybrids. We observed ssDNA patches in association with R-loop formation, however, they occur further upstream from the R-loops and are present at a slightly higher frequency to that of ssDNA patches amplified using non-converted primers for the 5′Sμ region (Figure 5D; Cont vs. Cont with R-loop and hRH1 vs. hRH1 with R-loop). Interestingly, the reduction in R-loop length and frequency caused by RNaseH1 did not lead to a decrease in CSR to IgA (Figure 5E). However, it is important to note that R-loops are still produced in the RNaseH1 expressing cells, and it is possible that this reduction in R-loop lengths and frequency is not sufficient to alter CSR activity. Furthermore, RNaseH1 expression did not impact the SHM frequency or the strand bias of mutations within the 5′Sμ region in CH12F3-2 cells (Figure 5F). In addition, expression of RNaseH1 in Ramos cells did not impact the SHM frequency at the V-region (Figure S5I) or at the GFP gene (Figure S5G), nor did it impact the ssDNA frequency at the V-region (Figure S5H). These data show that the bisulfite-accessible ssDNA patches are not caused by RNA-DNA hybrids, and suggest that under conventional circumstances, while the top strand of DNA is made accessible by the formation of R-loops at switch regions, the existence of ssDNA patches on both strands provides another mechanism by which AID can access the bottom strand of the switch region to mediate SHM and CSR. Thus, one of perhaps many mechanisms by which CSR proceeds is via the concerted action of R-loops, which allows access of AID to the top strand of DNA, and supercoiled-induced ssDNA patches, which allow access of AID to the bottom strand.

## Discussion

While it is accepted that AID mediates SHM and CSR by deaminating ssDNA within Ig genes, the nature of the ssDNA and how it is generated has remained elusive. In this report, we reveal that Ig genes and a non-Ig transgene that is mutated by AID are enriched for short ∼7 nucleotide ssDNA patches. These ssDNA patches are reactive to sodium bisulfite only in intact nuclei [18]. Indeed, the ssDNA patches described in this report are consistent with the parameters necessary for an *in vivo* AID substrate, namely that: (1) these patches are found on both DNA strands thereby providing an explanation for the unbiased strand activity of AID during both SHM and CSR [10], [11] (Figure S2D Figure 5F), (2) the size of these patches correspond with the preferred *in vitro* substrate size for AID [49], and (3) patch formation is dependent on transcription elongation, concurrent with the requirement of transcription for SHM [5], [6]. Furthermore, we found that the frequency of these ssDNA patches strongly correlates with SHM rates within the Ig V-region and within a non-Ig transgene (GFP) that is mutated by AID. Together these data not only provide better insight into the role of DNA accessibilty during SHM and CSR, they also provide a model and mechanism of formation for one of the possible *in vivo* substrates of AID deamination.

The Ig switch regions have been shown to adopt a structure whereby the nascent sense transcript from the RNA polymerase II complex forms a RNA:DNA hybrid (i.e. R-loop) with the bottom DNA strand. The locations of these R-loops correlate with CSR [24], [50] and a large deletion of the switch region reduces both R-loop formation and CSR [30]. In addition, inverting the γ1 switch region reduces CSR to γ1 by ∼3 fold, and this inversion is thought to reduce R-loop formation [51]. Thus, R-loops play an important role in producing ssDNA during CSR. However, while the R-loop model explains how the top strand becomes accessible to AID, on its own it fails to explain how the bottom strand would be targeted for deamination, a prerequisite for CSR, and thus other processes must be taking place to allow for AID to access the bottom strand. It was recently shown that sense transcription through the switch region is sufficient for CSR while antisense transcripts are dispensable for this process [52]. This suggests that secondary structures produced during sense transcription elongation provide access to AID on both strands. Indeed, ExoI excision tracts can also potentially expose ssDNA on the bottom strand during CSR [25]. Previous findings showed that the bottom strand in the Ig switch region does not harbour ssDNA [24], [53]. This result is most likely due to the method of bisulfite deamination, which was carried out on purified nucleic acid [24], [53]. By carrying out the sodium bisulfite assay on intact nuclei, we show that the region immediately upstream of the μ switch region harbours short ssDNA patches on both DNA strands that increase in frequency when primary B cells (Figure 2C) or CH12F3-2 cells (Figure 5D) are stimulated to undergo CSR. This assay also identifies long stretches of sodium-bisulfite conversion within switch regions in murine B cells [18] or CH12F3-2 cells (Figure 5B and 5C) which are likely caused by R-loop formation. Although the ssDNA patches are shorter (∼7 nucleotides) within the switch region than the observed ssDNA within R-loops that can be kilobases in length [24], the frequency of these patches are significantly higher (∼1 patch per kilobase within the 5′μ switch region) than the frequency of R-loops in primary murine B cells (4% of switch regions contain R-loops) [30]. Furthermore, since R-loops only form in G-rich sequences [24], rare A:T rich switch sequences that are enriched for AID hotspot motifs, such as in *Xenopus laevis*, are unlikely to adopt an R-loop configuration, but nonetheless support CSR [54] indicating that they are accessed by AID through an unknown mechanism. In scenarios where R-loops are either decreased or absent, we suggest that ssDNA patches that are found on both DNA strands are sufficient to produce the AID-initiated staggered dsDNA breaks associated with CSR events. In addition, it is also possible that an AID induced mutation leads to Exo1-mediated excision of the top strand exposing the bottom strand to AID-attack [25]. In the context of normal R-loop formation, we suggest that all of these processes cooperate to allow AID access to the non-template (top) and template (bottom) DNA strands.

It has been appreciated for some time that transcription of the Ig gene is required for both SHM and CSR processes [5], [6]. It was assumed that transcription produces the ssDNA necessary for AID reactivity [7], [15], [16], [32]. Our findings that transcription initiation [18] and elongation inhibitors ablate ssDNA patches in the V-region in Ramos cells and the 5′μ switch region in CH12F3-2 cells (Figure 1H and Figure 2D, respectively) and that nontranscribed genes contain no ssDNA patches (Figure 2D; CD4 gene) provides additional independent evidence that the short ssDNA patches observed in Ig sequences are transcription-dependent. However, it is unlikely that the ssDNA patches are produced by bisulfite conversion of ssDNA tracts within the RNA polymerase II transcription bubble itself. First, the ssDNA patches observed in this report are shorter than the predicted transcription bubble size of ∼11 nucleotides [19]. Second, a recent report shows that the RNA polymerase II complex transcribes the Ramos V-region only in the sense direction [23] which would lead to ssDNA formation on the top strand only since the nascent transcript is expected to protect the bottom strand from bisulfite conversion. However, ssDNA patches are observed on both strands at approximately equal frequency in the Ramos V-region [11] arguing against the transcription bubble as the source of ssDNA. Moreover, it is unlikely that AID can gain access to dC within the transcription bubble since it is largely occupied by the RNA polymerase II complex [19], [20]. In the context of the AID targeting factor Spt5, crystallographic studies of Spt5 complexed to RNA polymerase II show that ssDNA is buried within the active centre cleft of the transcription machinery [21], [22].

DNA supercoiling caused by transcription is a potential explanation for the ssDNA patches that we observe in Ig genes. Indeed, we observe an alteration in ssDNA frequencies in mammalian cells treated with the topoisomerase I inhibitor camptothecin, which can alter local DNA superhelicity. Furthermore, TopA-deficiency in *E.coli* results in hyper-negative supercoiling of transcribed plasmid DNA, increased frequency of ssDNA patches, and AID mutagenic activity (Figure 4). It is known that TopA-deficiency can lead to R-loop formation upon induction of transcription. However, R-loop formation does not occur during transcription induced negative supercoiling in a plasmid system when the nascent mRNA is translated [55]. In our system, AID is actively transcribed and translated upon IPTG induction. In addition, TopA-deficiency did not lead to increased ssDNA patch lengths which would be expected if these were R-loops (Figure 4C). Furthermore, we did not observe an increase in ssDNA patches on the top strand in the TopA-deficient clones (Figure S4B), which would be expected since the sense strand would be displaced by the transcript. These data are consistent with the notion that negative supercoiling is the likely source of the ssDNA patches.

Further support for negative supercoiling as the source of ssDNA patches that we observed is that AID and sodium bisulfite can deaminate supercoiled plasmid DNA but not relaxed linearized DNA *in vitro* [26]. During transcription, negative supercoiling develops upstream of the transcription complex [40], [41] and has been associated with melted DNA that can in turn lead to the formation of secondary DNA structures, such as stem loops and cruciforms [42], [43]. In contrast, positive supercoiling occurs downstream of the transcription complex [40]. The dual effects of positive and negative supercoiling may work in concert to increase targets for AID. That is, as the transcription complex progresses through the gene leaving in its wake under wound and melted DNA, positive supercoiling downstream of the transcription complex may act to slow down or pause RNA polymerase II. Indeed, Canugovi *et al.* observed that inducing pausing/stalling of T7 RNA polymerase resulted in the accumulation of multiple clustered AID-induced mutations *in vitro* [56], and AID was recently found to interact with Spt5, a factor associated with stalled RNA polymerase II [3]. Rajagopal *et al.* recently showed that RNA polymerase II complexes pause and accumulate upstream of the μ switch region [57], which might serve to provide DNA structures that act as targets for AID during CSR. Indeed, the slight increase in ssDNA patch frequency that we observed in sequences that contained R-loops in the CH12F3-2 cells (Figure 5D) correlates nicely with the findings of Rajagopal *et al.* [57] which showed an accumulation of RNA pol II just 5′ of the Sμ region, possibly due to R-loop formation. Furthermore, Wang *et al.* have shown histone marks at the switch regions indicative of open and accessible chromatin and this finding was also associated with RNA polymerase II presence and stalling at switch regions [58]. Stalling of the RNA polymerase II may not only result in the production and maintenance of R-loops, but may act to sustain secondary structures in the DNA produced by negative supercoiling. These findings suggest that the activity of AID on short ssDNA patches would largely be limited by the activity of topoisomerases, removing transcription-induced supercoiling and thus eliminating ssDNA patches for AID to act on. Indeed, Kobayashi *et al.* showed that topoisomerase 1 mRNA and protein levels were reduced upon AID expression and this reduction was associated with altered DNA structure at the μ switch region, increased switch region cleavage, and increased CSR [37]. The reduction in topoisomerase 1 may therefore lead to transcriptional pausing allowing for the increased duration of negatively supercoiled DNA that can be mutated by AID. On the other hand, complete inhibition of topoisomerase 1 by camptothecin might indirectly lead to the cessation of all RNA polymerase II transcription as the RNA polymerase may not be able to bypass the complex or lesion produced by the camptothecin thereby resulting in a reduction in ssDNA patches (Figure 2D) and AID-induced DNA breaks [37]. Another potential source for the generation of these ssDNA patches is the RNA exosome which was recently reported to associate with AID and stimulate AID activity to both DNA strands in a manner that is independent of replication protein A (RPA) or the phosphorylation status of AID [4]. Our current and previous findings [18] that the immunoglobulin genes are enriched for ssDNA patches on both strands is consistent with the activity proposed by the RNA exosome. Nevertheless, our current findings support the role of negative supercoiling in the generation of these patches, but do not preclude the involvement of the RNA exosome or the Spt5 factor. Future work will reveal whether these factors are in part or in whole responsible for the generation of these ssDNA patches that are observed in this report. Furthermore, it is important to note that while we suggest that ssDNA patches observed in this report are produced by transcription-induced negative supercoiling, ssDNA could be produced by other mechanisms, such as transcription-induced G4 DNA formation [59], melting of DNA during replication, interaction of transcription factors with DNA, and DNA repair intermediates [25].

While the evidence supports the notion that the bisulfite-accessible ssDNA patches that are observed in Ig genes are substrates for AID, their frequency is likely not the sole determinant of mutability. First, while the 5′ end of the V-region is enriched in ssDNA patches (Figure 1A), it does not harbour many mutations (Figure S2D). Thus, near the V-region promoter, there is poor correlation between ssDNA patches and mutation frequency. In fact, previous studies have shown that the region near the promoter is spared from mutation (e.g. [60]) for reasons that are not known. Our data clearly states that it is not because there are no ssDNA patches there, and hence there must be another reason for this result. Perhaps the explanation is that AID associates with the elongation RNA polymerase II complex, or AID associates with stalled RNA polymerases, and both of these don't occur near the promoter region. Second, non-mutating genes also harbour ssDNA patches (Figure 3). Rather, our work suggests that ssDNA patches, which occur in transcribed genes and are produced by negative supercoiling, render DNA single stranded and accessible to AID, however some other molecular feature is required to target AID to Ig genes to mediate SHM and CSR. Thus, B cells have likely evolved several mechanisms to ensure enhanced targeting of AID to Ig genes. It is likely that multiple conditions must be met in a gene in order to produce the potential for high mutagenic activity by AID; these include a high frequency of ssDNA, the presence of specific cis-acting sequences [61], [62], a high degree of transcriptional pausing [3], [56], association with the RNA exosome [4], and the association of AID to trans-acting factors which function to link AID to each of the above-mentioned conditions (e.g. transcriptional pausing and Spt5). Integration of these distinct targeting mechanisms would ensure that the Ig locus is preferentially mutated by AID over other genomic regions, while if any of these conditions on their own are met, it could subject that gene to low levels of AID activity [38], [63], [64].

## Materials and Methods

### Ethics Statement

All animal work was conducted according to our institutions animal welfare guidelines.

### In Vitro Cell Culture

Ramos and CH12F3-2 cells were cultured and stimulated as previously described [11], [46]. Retroviral transductions were carried out as previously described [65]. Ramos cells, CH12F3-2 cells and primary *ex vivo* LPS stimulated B cells were treated with inhibitors for 24 hours prior to *in situ* bisulfite treatment using their respective LD_50_ and LD_25_ values. The LD_50_ for actinomycin-D (Sigma-Aldrich) and camptothecin (Sigma-Aldrich) treatments were 10.85 ng/mL and 0.3405 µM for Ramos cells, respectively. LD_25_ for actinomycin-D and camptothecin were 6.4 ng/mL and 0.23 µM for Ramos cells, respectively. The LD_50_ for camptothecin treatment in CH12F3-2 cells was 0.75 µM. The LD50 for camptothecin treatment in primary *ex vivo* LPS stimulated B cells was 0.55 µM. LD_50_s for Ramos and CH12F3-2 cells were measured by trypan blue exclusion and confirmed by flow cytometry using Annexin V (eBioscience) staining 24 hours after drug was added. <1% DMSO was present at these inhibitor concentrations during culture. CH12F3-2 and Ramos cells were transfected with pA-HRH-delter-Zeo (see below) and selected with zeocin (Invitrogen) at 300 µg/ml. Western blots for RNaseH1 (Santa Cruz biotechnology; C-18) and β-actin (Abcam) were carried out using manufacturer's instructions. CSR assays using CH12F3-2 cells were described previously [46]. Lentiviral shRNA constructs for AID-targeting (TRC0000112031 and TRC0000112033) and GFP-targeting negative control (TRC0000072179) shRNA were provided by Dr. Jason Moffat. CH12F3-2 cells were transduced with lentivirus for 24 hours, followed by puromycin selection for 3 days. Positively transduced cells were then subjected to CSR assays.

### Bacterial Strains and Plasmids

The pGFP*I puro retroviral vector was kindly provided by Dr. Matthias Wabl [27]. The GFP gene harbours a nonsense codon within an AID hotspot motif. An AID-mediated nonsense reversion mutation will occur on the bottom strand within the dC that is opposite the dG in the TAG codon. The vector was linearized with XhoI and the 245 bp and 1.1 kb V–region promoters were introduced into the retroviral vector using primers with XhoI flanking regions. To amplify the promoter regions, the following primers were used: RevVprom with FwdVPS (245 bp promoter) and RevVprom with FwdVPL (1.1 kb promoter) (see Table S1). The vector encoding RNaseH1 (pA-HRH-delter-Zeo) was kindly provided by Dr. Xialu Li [48]. VS111(DE3) [F^−^ *LAM- rph-I* Δ*topA*] and MG1655 [F^−^ *LAM- rph-I*] were kindly donated by Dr. Fenfei Leng (Florida International University). Strain MG1655 was made into a DE3 strain using the Lambda DE3 Lysogenization Kit (EMD Cat# 69734-3). For the bisulfite analysis on the AID gene, BL21 (DE3) and VS111(DE3) were transformed with the IPTG-inducible plasmid p-GEX5.3-hAID(T27N) (catalytically inactive), and grown in Luria broth (LB) supplemented with 200 µg/mL ampicillin, while VS111(DE3) was additionally supplemented with 20 µg/mL chloramphenicol. IPTG-inductions were carried out for 1 hr at 1 µg/mL IPTG when cells reached an O.D. 600 of 0.4. For the kanamycin reversion assay, p-GEX5.3 and p-GEX5.3-hAID(WT) plasmids were used to introduce a kanamycin sensitive gene which contains an L94P mutation producing a p-GEX5.3KanS empty vector control and p-GEX5.3-hAID(WT)KanS plasmid respectively. The KanR gene has its own bacterial promoter and was placed directly downstream of the AID gene. There is no transcription terminator between the AID and KanR gene.

### In Situ Bisulfite Assay

*In situ* bisulfite assay on Ramos, CH12F3-2 and *ex vivo* mouse B cells was performed as previously described [18]. In situ bisulfite on bacteria was carried out on BL21(DE3) and VS111(DE3). Bacteria was grown to an optical density of 0.4 and supplemented with 1 mM IPTG for 1 hour after which a modified version of the *in situ* sodium bisulfite assay was carried out (a detailed protocol will be provided upon request). ssDNA patches were defined as lengths of DNA that contain at least 2 consecutively bisulfite-converted dCs on the same strand. Average patch lengths are reported in this study, which were obtained from averaging the minimum and maximum patch lengths for each individual patch. That is, the minimum length of ssDNA patches is the distance between the converted dCs (or converted dGs for the bottom strand). The maximum length of ssDNA is the distance between (and excluding) the nonconverted dCs (or nonconverted dGs for the bottom strand) extending out from the 5′ and 3′ ends of the ssDNA patch. Two ssDNA patches in uninduced BL21(DE3) occurred in A/T rich regions (Figure 4C) resulting in short minimum patches (i.e. 3 and 4 nucleotides) but very large maximum patch sizes (i.e. 19 and 22 nucleotides) owing to the lack of dC nucleotides next to the patch. In this case, we assumed a maximum patch size that would result in an average patch size that we observed in otherwise typical patches. We previously defined patches as those containing at least 3 consecutively bisulfite-converted dCs [18], and this number was chosen based on a statistically significant difference between ssDNA at the V- and constant regions which was not the case when 2 consecutively bisulfite-converted dCs was used to define patches. Hence, the average patch lengths reported in that study were longer (∼10 nucleotides). In this report, we defined patches as having 2 consecutively bisulfite-converted dCs since statistically significant differences in ssDNA frequency existed between the Ramos V- and non-V-regions (Figure S1A). The ssDNA frequency at each gene was calculated by dividing the total number of nucleotides found in ssDNA (average ssDNA patch lengths were used) by the total number of nucleotides analysed. Extracted genomic DNA from *in situ* bisulfite treated-CH12F3-2 clones were subjected to PCR amplification to determine R-loops using a forward primer that binds ∼800 bp upstream of the murine μ switch region (Table S1; FMusSmu) and a reverse primer that binds just 5′ of the μ switch region in which the dGs have been replaced by dAs (Table S1; RRloop) to bind to sequences in which the dCs have been converted to dUs on the top strand R-loop lengths were determined as contiguously converted dCs starting from the reverse primer and extending 5′ of that region.

### Flow Cytometric Analyses

Flow cytometry for GFP expression in Ramos cells was carried out as previously described [65]. Reversion of the stop codon at the Igμ gene in Ramos cells was carried out as previously described [11]. Flow cytometry on CH12F3-2 cells stimulated to switch were carried out using intracellular staining with PE-anti-mouse IgA antibody (eBioscience) and extracellular staining with PE-anti-mouse IgA antibody (Southern Biotech).

### Primary B Cell Stimulation

All animal work was conducted according to our institutions animal welfare guidelines. Primary mature B cells were isolated and stimulated with LPS to induce CSR to the IgG3 isotype, as previously described [66]. Briefly, primary B cells were isolated from spleen of 10–12 week old mice using a magnetic negative selection B-cell enrichment kit (Stem Cell Technologies). ∼1 to 2×10^7^ cells were seeded in a 10 cm culture dish and exposed to 25 µg/mL of lipopolysaccharide (*Escherichia coli* serotype 055:B5; Sigma-Aldrich) for 48 hours. Primary mature B cells were also stimulated to isotype switch to IgA with 2 ng/mL TGF-β, 1.5 ng/mL IL-5, 25 µg/mL LPS and 10 ng/mL IL-4 for 48 hours and to switch to IgG1 25 µg/mL LPS and 10 ng/mL IL-4 for 48 hours. Cultured cells were grown in RPMI 1640 medium (Invitrogen) supplemented with 10% fetal calf serum (HyClone) and 50 µM β-mercaptoethanol (Invitrogen) at 37°C and 5% CO_2_.

### Chloroquine Agarose Gel Analysis of Plasmid Topology

BL21(DE3) and VS111(DE3) bacterial strains expressing the catalytically inactive AID expression plasmid, p-GEX5.3-hAID(T27N), were grown to an optical density of 0.4 and induced for 1 hr with 1 mM IPTG as described above for Figure 4A. For Figure S4A BL21(DE3), VS111(DE3) and MG1655(DE3) strains expressing a control plasmid, p-GEX5.3KanS, were used. Following IPTG induction, plasmids were obtained using the GeneJet Plasmid mini prep kit (Fermentas). Plasmid topological status from each strain was analysed on a 1% agarose gel containing 5 µg/mL chloroquine (Sigma) run overnight at 3 V/cm. Gels were stained with 1 µg/ml ethidium bromide (BioShop) for 30 mins and exposed to visualize plasmid bands. Chloroquine is a DNA intercalator that introduces positive supercoils into DNA and allows for the resolution of different negatively supercoiled plasmid species. During chloroquine gel electrophoresis, more negatively supercoiled plasmids run further through the gel than their less negatively supercoiled counterparts.

### Kanamycin Reversion Assay

BL21, MG1655, and VS111 bacterial strains containing the p-GEX5.3-hAID(WT)KanS and control p-GEX5.3KanS constructs were grown in LB Ampicillin (200 µg/mL Ampicillin) to an OD 0.3. Samples were then induced with 0.5 mM IPTG for 1 hour at 18°C. OD values were obtained after 1 hour to determine live bacterial cell numbers. All bacteria were plated on LB Kanamycin plates (100 µg/mL Kanamycin). Plates were incubated O/N at 37°C and colonies were counted the following day. Kanamycin reversion frequencies were calculated by dividing the total number of Kanamycin resistant colonies by the total number of live bacteria plated out. To determine the AID-specific mutation frequency, the mutation frequency of control p-GEX5.3KanS was subtracted from the p-GEX5.3-hAID(WT)KanS mutation frequency for each strain.

### Sequencing of Ramos V-Region and CH12F3-2 5′Sμ Region for Mutation Analysis

Genomic DNA was prepared and regions of interest were amplified using Pfu Ultra II (Agilent). Amplification of the Ramos V-region was done using the Ramos V-region primers (Table S1: Ramos V-region). Amplification of the CH12F3-2 5′Sμ region was done using the Murine 5′μ Switch primers (Table S1: Murine 5′μ Switch). PCR products were cloned and sequenced. For Figure S5I, 11519 nucleotides and 10865 nucleotides were examined for the Ramos V-region in vector control and hRH1 expressing cells, respectively. For Figure 5F, 16029 nucleotides and 24781 nucleotides were examined for the CH12F3-2 5′Sμ region in control and hRH1 expressing clones respectively.

### qPCR Analysis

qPCR analysis of GFP expression relative to GAPDH expression was done on Ramos clone GFP-14 and VPL-6. Total RNA was isolated using TRIzol reagent (Invitrogen) as per manufacturer's protocols. Reverse transcription was conducted using an oligo dT primer and SuperScriptIII (Invirtogen) as per manufacturer's protocols. qPCR was conducted on the 7300 Real Time PCR System (Applied Biosystems) using primers (Table S1) for GFP qPCR and GAPDH qPCR.

### Statistical Analysis

All analyses were performed using GraphPad Prism. For Student's t tests, Mann-Whitney tests and linear regression analysis, *P* values of 0.05 or less were considered significant. All error bars represent the standard error of the mean (SEM).

## Supporting Information

Figure S1 ssDNA Frequencies at 3 Regions within the Ig Locus in AID-Sufficient and AID-Deficient Ramos cells. A) ssDNA frequencies at distinct regions within the Ig locus. SHM is restricted to the V-region while the V promoter and 5′μ switch regions in Ramos are not mutated. Statistical analyses were performed using the Student's t-test (* = P = 0.0368 comparing ssDNA frequencies at V promoter to V-region; * = P = 0.0104 comparing ssDNA frequencies at 5′μ switch to V-region). B) ssDNA frequencies at the V-region in AID high-expressing Ramos 7 (R7) and AID negative Ramos 1 (R1) clones. ssDNA frequency is defined as the total number of nucleotides found within a ssDNA patch divided by the total number of nucleotides sequenced.
(TIF)

Figure S2 Mutation Frequencies of a GFP Proviral Transgene in Different Ramos Cell Transductants. A) Schematic showing retroviral constructs (GFP, VPS GFP and VPL GFP) that were used to transduced Ramos cells. Grey box represents a sequence of the V-region promoter (245 bp for VPS and 1.1 kb for VPL constructs) that was inserted into the retroviral construct. TAG represents the nonsense codon present in the GFP gene that when reverted, allows for GFP reversion/expression analysis by flow cytometry. B) GFP reversion frequencies observed in individual Ramos 1 clones (R1: AID-negative) and Ramos 7 clones (R7: AID-high) transduced with retroviruses harbouring different GFP constructs. Values for GFP mutation frequencies in R1 were obtained from [65]. Lines represent median GFP revertant frequencies. Statistical analysis was performed using the Mann-Whitney test (* = P = 0.0267). C) Fluctuation analyses carried out on individual clones. ∼10 subclones for 4 clones were analyzed for each GFP proviral construct. Lines represent the median GFP revertant frequencies. D) Mutation analysis of the Ramos V-region. Mutations at dC (open triangles) and at dA (grey triangles) are depicted. The normalized dC mutation frequency on the top strand of 0.48 (63 mutations/131 C present on the top strand) over the normalized dC mutation frequency of the bottom strand of 0.44 (67 mutations/151 C present on the bottom strand) shows no strong strand bias of mutations.
(TIF)

Figure S3 ssDNA Patch Strand Bias Analysis and ssDNA Frequencies at S-Regions Induced to Undergo CSR in *ex vivo* Mouse B Cells. A) ssDNA patch strand bias as expresses as ssDNA frequencies of the top strand divided by the total ssDNA frequency for the 3′JH4 and 5′Sμ in unstimulated and LPS stimulated *ex vivo* mouse B cells. A value of 0.5 represents no strand bias. B) ssDNA frequencies present at the CD4 gene (white bar), 5′Sγ1 in unstimulated, LPS stimulated and LPS+IL-4 stimulated B cells (light grey bars), 5′Sγ3 in unstimulated and LPS stimulated B cells (medium grey bars) and 5′Sα in unstimulated and LPS, IL-4, IL-5+TGF-β stimulated B cells (dark grey bars). Percentages above bars represent the percent of isotype switched B cells.
(TIF)

Figure S4 ssDNA Strand Bias and Plasmid Topology Analysis in *E.coli*. A) Plasmid topology was determined for uninduced and 1 hr IPTG-induced bacterial strains by chloroquine agarose gel analysis for plasmids derived from wildtype BL21(DE3), MG1655(DE3) and TopA deficient VS111(DE3) bacterial strains. Highly-negative supercoiled DNA (HNSC) runs faster than its less supercoiled counterpart. B) ssDNA patch strand bias as expressed as ssDNA frequency on the top strand divided by the total ssDNA frequency for uninduced and IPTG-induced BL21 and VS111 strains. C) Western blot for AID expression in BL21, MG1655 and VS111 strains containing the pGEX5.3-hAID(WT)KanS plasmid in uninduced or IPTG-induced bacterial strains.
(TIF)

Figure S5 The Effects of RNaseH1 Expression During CSR in CH12F3-2 cells and SHM in Ramos Cells. A) CH12F3-2 cells transduced with 2 different shRNA vectors that target AID, namely AID shRNA-1 and AID-shRNA-2 that knockdown AID protein by 5.8-fold and 2.4-fold, respectively, and B) inhibit CSR to IgA by 5-fold (** = p = 0.005) and 2.5-fold (** = p = 0.009), respectively. Values for fold decrease for AID protein (A) and for CSR (B) are shown relative to control clone (i.e. GFP-shRNA). C) ssDNA patch lengths, locations and strand distribution identified in the 5′μ switch region in unstimulated (grey) and CSR-stimulated CH12F3-2 cells (black) and in hRH1-expressing CSH12F3-2 cells in unstimulated (hatched light-grey) and CSR-stimulated cells (hatched dark-grey). D) ssDNA patch length in control and RNaseH1 expressing CH12F3-2 cells. E) ssDNA patch strand bias as expressed as ssDNA frequencies on the top strand divided by total ssDNA frequencies for each CH12F3-2 clone. RNaseH1-expressing clones are designated by an R. F) Proliferation analysis of empty vector control and hRH1-expressing CH12F3-2 clones. G) Ramos clone GFP-14 transfected with an RNaseH1 expression vector (hRH1) or empty vector control (Control) with an analysis of GFP reversion frequency. Students T-test was utilized to show no significant difference in GFP reversion frequency in control and RNaseH1 expressing clones. White circles represent a mutation frequency of less than one in 10^6^ cells. H) ssDNA frequency of the V-region in Ramos clone GFP-14 control and hRH1 expressing cells. I) Mutation frequency of the Ramos V-region in Ramos clone GFP-14 control and hRH1 expressing cells.
(TIF)

Table S1 Primer Pairs Utilized in this Manuscript. Primer pairs for each of the different regions examined/amplified in this manuscript.
(DOC)
