# Supplementary figures and images for: Negative Supercoiling Creates Single-Stranded Patches of DNA That Are Substrates for AID–Mediated Mutagenesis
Source: PLoS Genet. 2012 Feb 9;8(2):e1002518. doi: 10.1371/journal.pgen.1002518 (PMC3276561; doi:10.1371/journal.pgen.1002518)

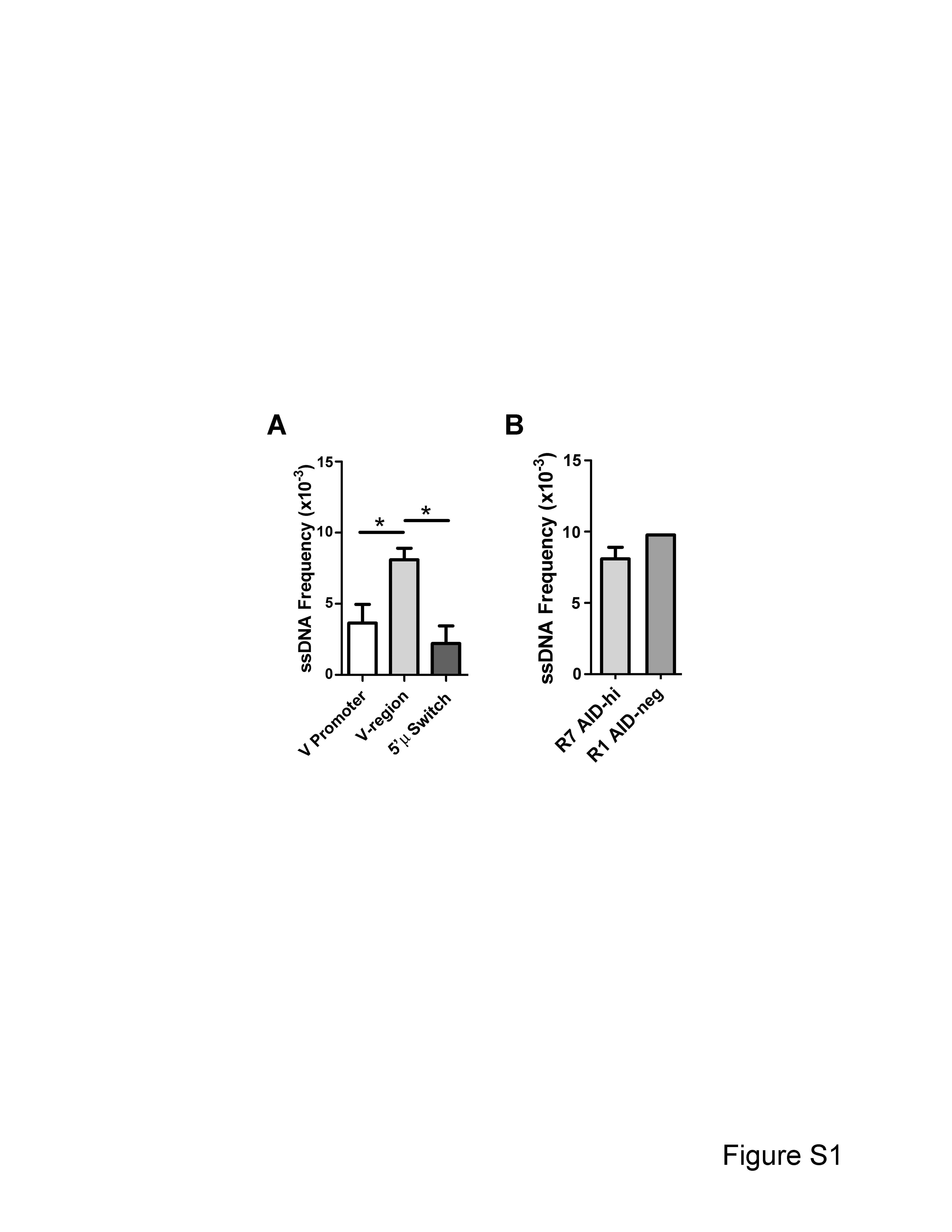

Supplement: Figure S1 — ssDNA Frequencies at 3 Regions within the Ig Locus in AID-Sufficient and AID-Deficient Ramos cells. A) ssDNA frequencies at distinct regions within the Ig locus. SHM is restricted to the V-region while the V promoter and 5′μ switch regions in Ramos are not mutated. Statistical analyses were performed using the Student's t-test (* = P = 0.0368 comparing ssDNA frequencies at V promoter to V-region; * = P = 0.0104 comparing ssDNA frequencies at 5′μ switch to V-region). B) ssDNA frequencies at the V-region in AID high-expressing Ramos 7 (R7) and AID negative Ramos 1 (R1) clones. ssDNA frequency is defined as the total number of nucleotides found within a ssDNA patch divided by the total number of nucleotides sequenced. (TIF) [file pgen.1002518.s001.tif]

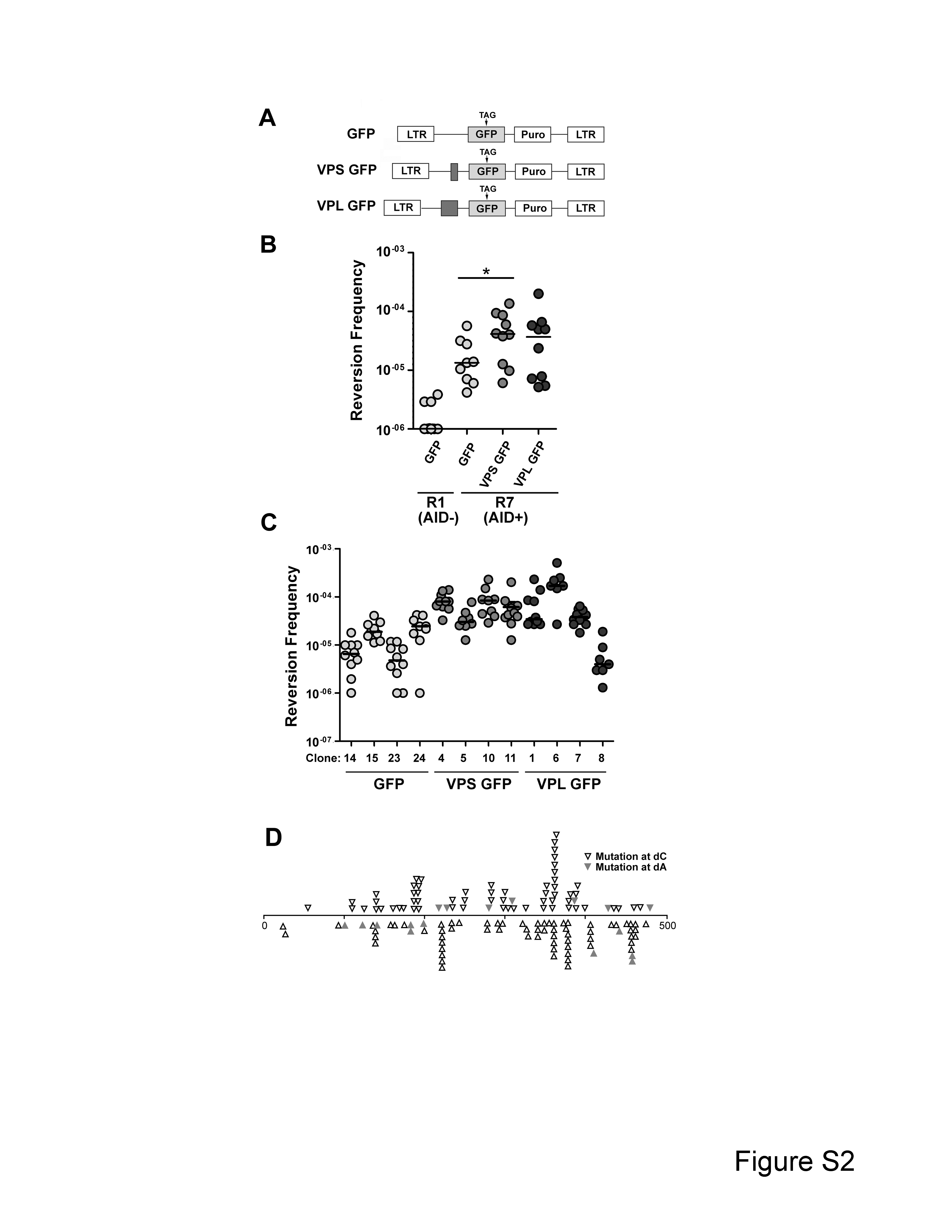

Supplement: Figure S2 — Mutation Frequencies of a GFP Proviral Transgene in Different Ramos Cell Transductants. A) Schematic showing retroviral constructs (GFP, VPS GFP and VPL GFP) that were used to transduced Ramos cells. Grey box represents a sequence of the V-region promoter (245 bp for VPS and 1.1 kb for VPL constructs) that was inserted into the retroviral construct. TAG represents the nonsense codon present in the GFP gene that when reverted, allows for GFP reversion/expression analysis by flow cytometry. B) GFP reversion frequencies observed in individual Ramos 1 clones (R1: AID-negative) and Ramos 7 clones (R7: AID-high) transduced with retroviruses harbouring different GFP constructs. Values for GFP mutation frequencies in R1 were obtained from [65]. Lines represent median GFP revertant frequencies. Statistical analysis was performed using the Mann-Whitney test (* = P = 0.0267). C) Fluctuation analyses carried out on individual clones. ∼10 subclones for 4 clones were analyzed for each GFP proviral construct. Lines represent the median GFP revertant frequencies. D) Mutation analysis of the Ramos V-region. Mutations at dC (open triangles) and at dA (grey triangles) are depicted. The normalized dC mutation frequency on the top strand of 0.48 (63 mutations/131 C present on the top strand) over the normalized dC mutation frequency of the bottom strand of 0.44 (67 mutations/151 C present on the bottom strand) shows no strong strand bias of mutations. (TIF) [file pgen.1002518.s002.tif]

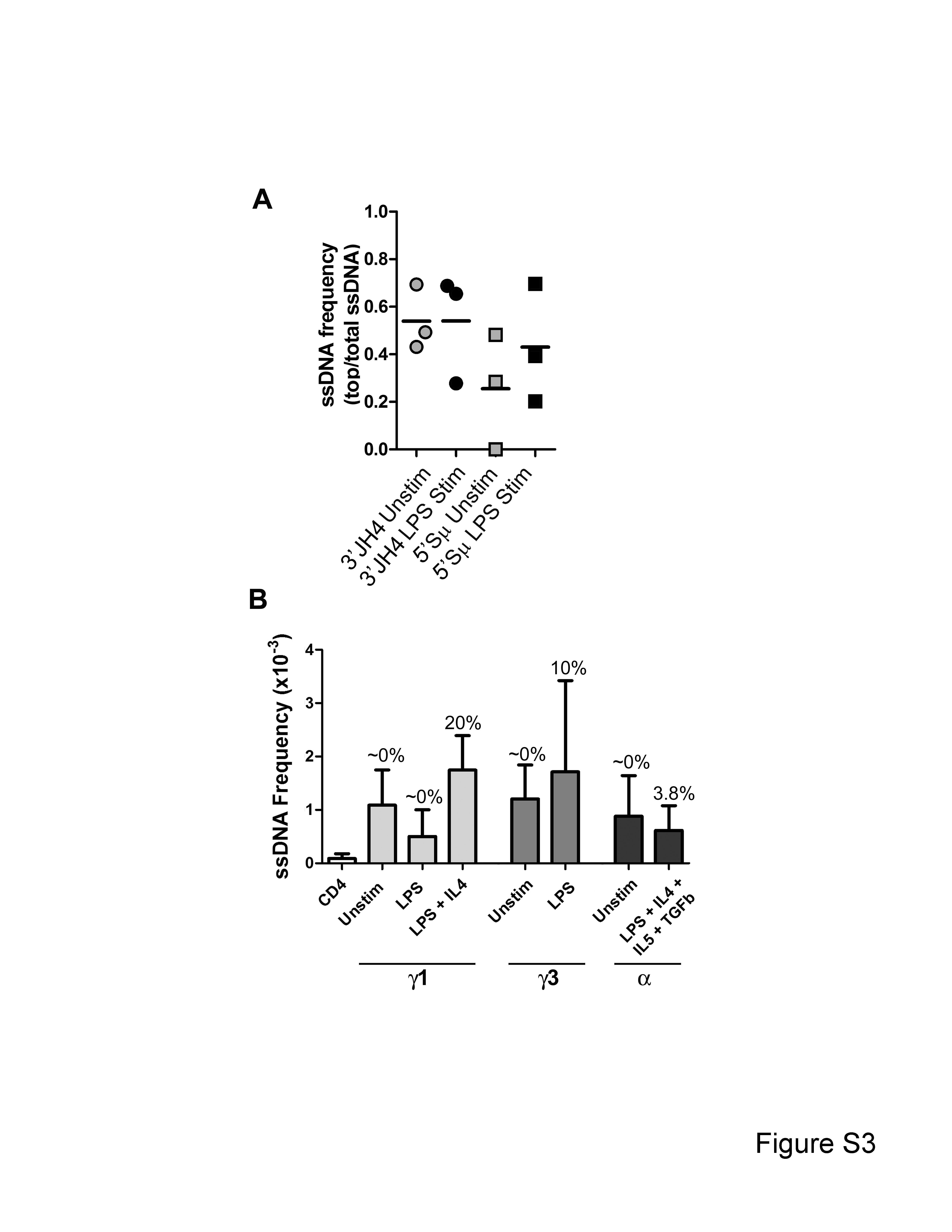

Supplement: Figure S3 — ssDNA Patch Strand Bias Analysis and ssDNA Frequencies at S-Regions Induced to Undergo CSR in ex vivo Mouse B Cells. A) ssDNA patch strand bias as expresses as ssDNA frequencies of the top strand divided by the total ssDNA frequency for the 3′JH4 and 5′Sμ in unstimulated and LPS stimulated ex vivo mouse B cells. A value of 0.5 represents no strand bias. B) ssDNA frequencies present at the CD4 gene (white bar), 5′Sγ1 in unstimulated, LPS stimulated and LPS+IL-4 stimulated B cells (light grey bars), 5′Sγ3 in unstimulated and LPS stimulated B cells (medium grey bars) and 5′Sα in unstimulated and LPS, IL-4, IL-5+TGF-β stimulated B cells (dark grey bars). Percentages above bars represent the percent of isotype switched B cells. (TIF) [file pgen.1002518.s003.tif]

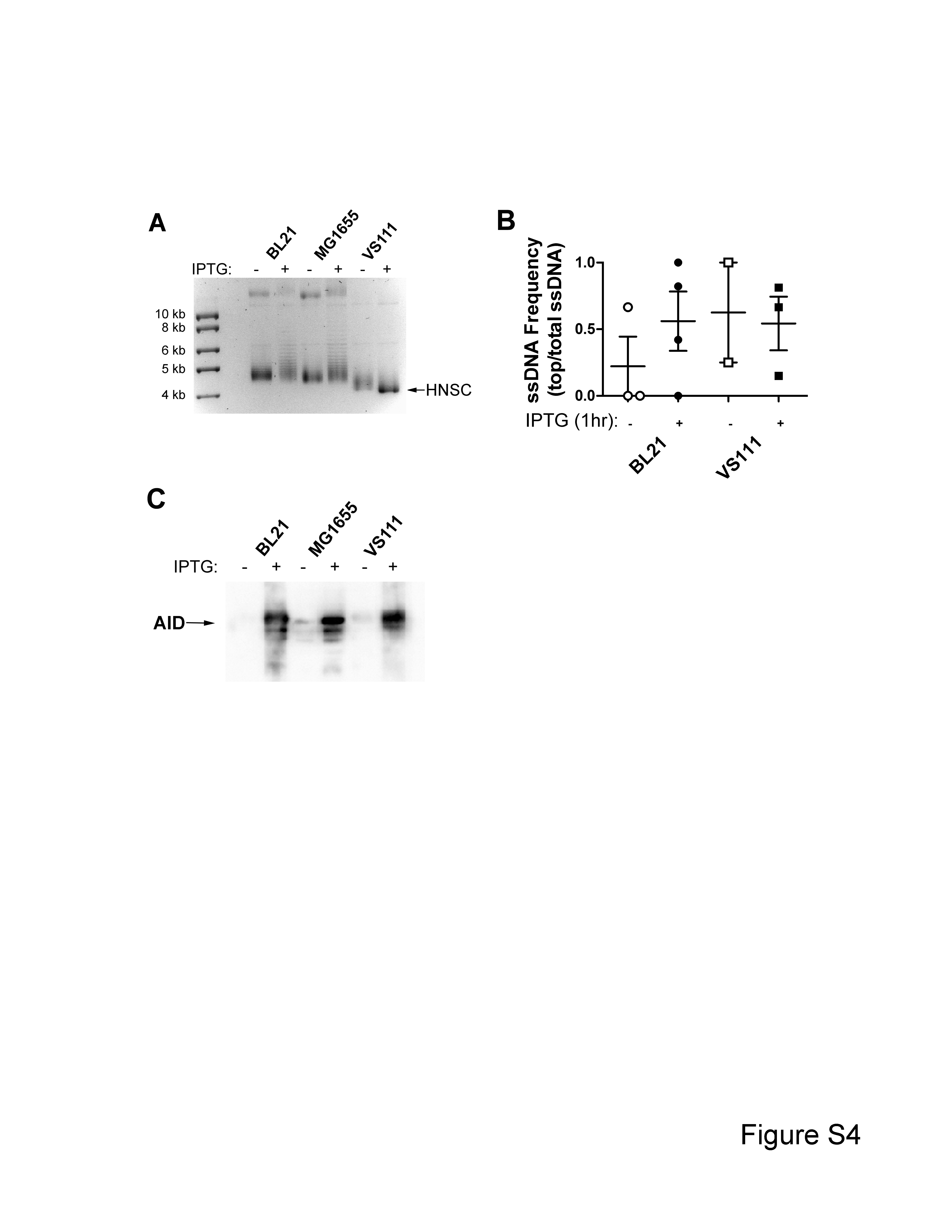

Supplement: Figure S4 — ssDNA Strand Bias and Plasmid Topology Analysis in E.coli. A) Plasmid topology was determined for uninduced and 1 hr IPTG-induced bacterial strains by chloroquine agarose gel analysis for plasmids derived from wildtype BL21(DE3), MG1655(DE3) and TopA deficient VS111(DE3) bacterial strains. Highly-negative supercoiled DNA (HNSC) runs faster than its less supercoiled counterpart. B) ssDNA patch strand bias as expressed as ssDNA frequency on the top strand divided by the total ssDNA frequency for uninduced and IPTG-induced BL21 and VS111 strains. C) Western blot for AID expression in BL21, MG1655 and VS111 strains containing the pGEX5.3-hAID(WT)KanS plasmid in uninduced or IPTG-induced bacterial strains. (TIF) [file pgen.1002518.s004.tif]

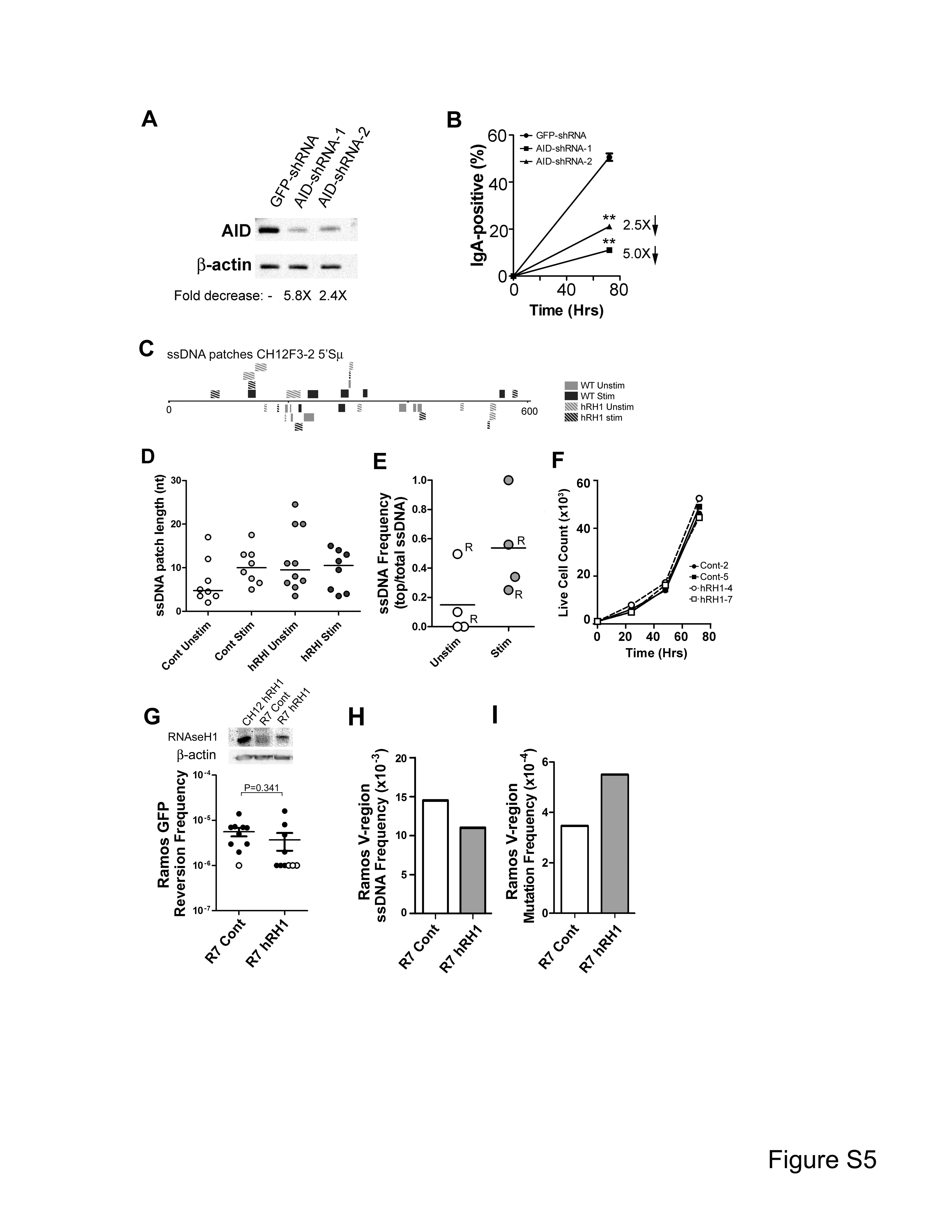

Supplement: Figure S5 — The Effects of RNaseH1 Expression During CSR in CH12F3-2 cells and SHM in Ramos Cells. A) CH12F3-2 cells transduced with 2 different shRNA vectors that target AID, namely AID shRNA-1 and AID-shRNA-2 that knockdown AID protein by 5.8-fold and 2.4-fold, respectively, and B) inhibit CSR to IgA by 5-fold (** = p = 0.005) and 2.5-fold (** = p = 0.009), respectively. Values for fold decrease for AID protein (A) and for CSR (B) are shown relative to control clone (i.e. GFP-shRNA). C) ssDNA patch lengths, locations and strand distribution identified in the 5′μ switch region in unstimulated (grey) and CSR-stimulated CH12F3-2 cells (black) and in hRH1-expressing CSH12F3-2 cells in unstimulated (hatched light-grey) and CSR-stimulated cells (hatched dark-grey). D) ssDNA patch length in control and RNaseH1 expressing CH12F3-2 cells. E) ssDNA patch strand bias as expressed as ssDNA frequencies on the top strand divided by total ssDNA frequencies for each CH12F3-2 clone. RNaseH1-expressing clones are designated by an R. F) Proliferation analysis of empty vector control and hRH1-expressing CH12F3-2 clones. G) Ramos clone GFP-14 transfected with an RNaseH1 expression vector (hRH1) or empty vector control (Control) with an analysis of GFP reversion frequency. Students T-test was utilized to show no significant difference in GFP reversion frequency in control and RNaseH1 expressing clones. White circles represent a mutation frequency of less than one in 106 cells. H) ssDNA frequency of the V-region in Ramos clone GFP-14 control and hRH1 expressing cells. I) Mutation frequency of the Ramos V-region in Ramos clone GFP-14 control and hRH1 expressing cells. (TIF) [file pgen.1002518.s005.tif]
