# Supplementary material for: Negative Supercoiling Creates Single-Stranded Patches of DNA That Are Substrates for AID–Mediated Mutagenesis
Source: PLoS Genet. 2012 Feb 9;8(2):e1002518. doi: 10.1371/journal.pgen.1002518 (PMC3276561; doi:10.1371/journal.pgen.1002518)
Supplement: Table S1 — Primer Pairs Utilized in this Manuscript. Primer pairs for each of the different regions examined/amplified in this manuscript. (DOC) [file pgen.1002518.s006.doc]

| **Region amplified** | **Forward primer** | **Reverse Primer** |
| --- | --- | --- |
| VPS Promoter | FwdVPS: 5’- gtttactcgagtgggtttggtgaggggagg-3’ | RevVprom: 5’- ctttactcgagtgttcttgtgcaggaggtc-3’ |
| VPL promoter | FwdVPL: 5’-gtttactcgagagagagcccaggtttatcc-3’ | RevVprom: 5’- ctttactcgagtgttcttgtgcaggaggtc-3’ |
| Ramos V promoter | FVp: 5’-gtttactcgagtgggtttggtgaggggagg-3’ | RVp: 5’-ctttactcgagtgttcttgtgcaggaggtc-3’ |
| Ramos V-region | FRamV: 5’-aaaagctagcacaagaacatgaaacacc-3’ | RRamV: 5’-gcggtacctgaggagacggtgacc-3’ |
| Ramos 5’μ Switch | FRamSw: 5’-gtaggtcatcatcgcaccc-3’ | RRamSw: 5’-gtctcagctaaagccatctc-3’ |
| GFP transgene | FGFP: 5’-ggggtaccatggtgagcaagggcg-3’ | RGFP: 5’-ctagatctttacttgtacagctcgtcc-3’ |
| Murine V-region | FMusV: 5’-ggaattcgcctgacatctgaggactctgc-3’ | RMusV: 5’-gactagtcctctccagtttcggctgaatcc-3’ |
| Murine 5’μ Switch | FMusSmu: 5’-acccaggctaagaaggcaatcc-3’ | RMusSmu: 5’-caccccaacacagcgtagcata-3’ |
| Murine 5’γ1 Switch | FMusSg1: 5’-ttaggagtcaatctcagtgc-3’ | RMusSg1: 5’-cagttttctgagctcctaag-3’ |
| Murine 5’γ3 Switch | FMusSg3: 5’-tgtgaagtaccagaatctgag-3’ | RMusSg3: 5’-tattctatacttccacctaccc-3’ |
| Murine Fas | FFas: 5’-ctttcgctcttggctaagtg-3’ | RFas: 5’-atagccctggtcgagtctg-3’ |
| Murine Trp53 | FTrp53: 5’-gttcattgggaccatcctggctgt-3’ | RTrp53: 5’-cggaatgcgttaagcaagggaat-3’ |
| Murine PCNA | FPCNA: 5’-ttggtagttgtcgctgtaggc-3’ | RPCNA: 5’-ccatttcacactcacggttg-3’ |
| Murine Lyn | FLyn: 5’-ctatcccgtcgcgtgaacag-3’ | RLyn: 5’-gagagaatgcagggcctagat-3’ |
| Murine 5’Sµ R-loop | FMusSmu: 5’-acccaggctaagaaggcaatcc-3’ | RRloop: 5’-caacacaacataacataactaaa-3’ |
| Murine CD4 | FCD4: 5’-aggcaagcgctctactgtgaac-3’ | RCD4: 5’-atgccctgtaatctgaaaactcg-3’ |
| Human AID | FhAID: 5’-gaggcaagaagacactctgg-3’ | RhAID: 5’-gtgacattcctggaagttgc-3’ |
| GFP qPCR | FqPCRGFP: 5’-ggagcgcaccatcttcttca-3’ | RqPCRGFP: 5’-agggtgtcgccctcgaa-3’ |
| Human GAPDH qPCR | FqPCRhGAPDH: 5’-aatcccatcaccatcttcca-3’ | RqPCRhGAPDH: 5’-tggactccacgacgtactca-3’ |

**Supplementary Table 1: Primer pairs used in this manuscript.**
